# Keeping it local: how CD8 TRMs regulate viral and cancer immunity

**DOI:** 10.3389/fimmu.2025.1618700

**Published:** 2025-12-17

**Authors:** Luiz Rodrigues Junior, Cristina Bonorino, Alisson Felipe Haubert, Marvin Paulo Lins, Gabriel Pozo Pereira, Pedro R. Torres Romao, Barry T. Rouse

**Affiliations:** 1 Laboratório de Imunovirologia, Universidade Federal de Ciências da Saúde de Porto Alegre (UFCSPA), Porto Alegre, Brazil; 2 Programa de Pós-Graduação em Biociências, UFCSPA, Porto Alegre, Brazil; 3 Programa de Pós-Graduação em Patologia, UFCSPA, Porto Alegre, Brazil; 4 Laboratório de Imunologia, Universidade Federal de Mato Grosso, Cuiaba, Brazil; 5 Department of Pathobiology, College of Veterinary Medicine, The University of Tennessee, Knoxville, TN, United States

**Keywords:** CD8, TRM, virus, cancer, lymphocyte

## Abstract

The induction of immune responses in tissues and mucosa has emerged as one of the most promising strategies for the development of more effective vaccines and immunotherapies. In this context, CD8^+^ resident memory T cells (CD8^+^ TRM) have arisen as key players in local immune surveillance, acting persistently within non-lymphoid tissues. These cells represent a new and promising frontier in local immune responses and as potential clinical tools. CD8^+^ TRM are being extensively investigated as therapeutic targets against viral infections and cancer, although their clinical applications have yet to be fully established. Understanding the molecular signals that regulate their generation, differentiation, maintenance, and activation is crucial for the precise targeting of their immune functions. This review explores the main mechanisms involved in the formation and maintenance of CD8^+^ TRM, from the strength of MHC: TCR interactions to the coordinated role of cytokines, chemokines, and transcription factors in tissue retention and the expression of markers such as CD69, CD103, and CD49a. By integrating this knowledge, we discuss strategies to manipulate these pathways with the goal of developing more effective vaccines and personalized therapies based on resident memory T cells. We also examine how these molecular signals and pathways, either independently or in combination, can be explored both in the fight against viral infections and cancer, and in identifying CD8^+^ TRM predictive biomarkers for response to anticancer immunotherapies across various tumor types.

## 1 Introduction

The idea that protection against a disease could be induced by inhaling ground up scabs from those that recovered from smallpox came from the Chinese more than 1000 years ago (1). This might be construed as the dawn of mucosal immunology. However, a more mechanistic understanding of local immunity was established in the 1960 with the demonstration that local immunization induced a unique immunoglobulin type referred to a secretory IgA (2). Subsequent studies showed that the greater efficacy of the oral Sabin polio vaccine to protect against enteric poliovirus infection was explained by its ability to induce secretory IgA at mucosal sites, which was absent following immunization with inactivated Salk vaccines (3). These observations on mucosal immunity focused on antibody mediated immune protection, but as we now know, the resolution of many infections and some cancers is carried out mainly by components of cellular immunity, particularly T cells.

Resident memory T (TRM) cells were initially identified as a heterogeneous population, both in terms of surface molecule expression and functional properties. Further complexity was revealed when it was demonstrated that T cells residing at tissue entry sites can be distinguished from memory T cells in systemic and lymphoid compartments based on membrane markers, transcriptional profiles, and cytokine responsiveness (4–6). Multiple studies have provided evidence that the signals governing TRM generation, maintenance, and function can vary not only between different tissues but also between distinct sites within the same tissue. This highlights the complexity and tissue-specific nature of the TRM differentiation program (7, 8). For instance, skin and intestinal TRMs heavily depend on TGF-β for their development and maintenance. In the intestine, however, an additional pathway involving retinoic acid signaling also contributes to TRM generation. While skin TRMs primarily rely on both TGF-β and retinoic acid, intestinal TRMs can utilize either factor. Nonetheless, when TGF-β is available, it remains the dominant driver of intestinal TRM formation (9). These findings underscore that TRM differentiation is governed by intricate, context-dependent molecular cues that are still being elucidated, particularly regarding how local signals and T cell receptor (TCR)-mediated pathways cooperate to define their activation and persistence.

The concept of TRM cells originated from seminal studies demonstrating that non-migratory T cells persist long-term within peripheral tissues following herpes simplex virus (HSV) skin infection, as shown through transplantation experiments (10). Similarly, intestinal graft models revealed that memory T cells embedded within the small intestinal epithelium remain locally confined and do not recirculate (11). Further insights came from parabiosis experiments, which showed that TRM cells not only persist at the initial site of infection but also disseminate throughout the entire skin surface, maintaining long-term residency (12). Phenotypically, TRM cells are defined by the expression of key integrins such as CD69, CD103, and CD49a, which orchestrate their retention and functional specialization within non-lymphoid tissues (13, 14). CD69 antagonizes sphingosine-1-phosphate receptor 1 (S1P_1_), thereby preventing tissue egress (15). CD103 (αE integrin) binds to E-cadherin on epithelial cells, promoting localization and anchorage within barrier tissues (16). CD49a (α1 integrin), which interacts with collagen IV, is associated with enhanced cytotoxic activity and long-term survival within tissue niches (17). Collectively, these surface molecules delineate the TRM phenotype and underlie their central functions, continuous immune surveillance and the rapid initiation of localized effector responses upon reinfection, thereby providing potent, site-specific protective immunity. Altogether, the combination of tissue residency and expression of adhesion and retention molecules defines the unique identity and immunological role of TRM cells.

Over the years, it has become evident that the tissue-surveilling capacity of TRM cells surpasses that of central memory (TCM) and effector memory (TEM) T cells in providing rapid and effective immunity at barrier sites. Studies using murine models of viral skin and lung infections demonstrated that TRM cells can promptly produce effector molecules locally, enabling them to respond more rapidly to pathogen re-exposure within the tissue compared with their circulating counterparts (12, 18). These findings have positioned TRMs as promising targets for immunotherapeutic interventions, particularly in cancer and chronic viral infections, and have also highlighted the need to evaluate vaccines based on their capacity to elicit tissue-resident responses. Nevertheless, several emerging new concepts in the TRM field require further evaluation before their clinical application becomes feasible. In this context, the present review examines the mechanisms involved in TRM induction and function across infection and cancer models, aiming to support the development of strategies with real translational potential.

Furthermore, this article provides a comprehensive synthesis of the molecular and transcriptional programs underlying CD8^+^ TRM differentiation. We link TCR signal strength with local cytokine and chemokine cues, illustrating how these pathways converge to shape TRM generation. By comparing antiviral and tumor settings, we reveal that similar molecular circuits govern TRM development in both contexts yet display remarkable plasticity according to the tissue microenvironment. A key contribution of this work is the discussion of how TCR–peptide–MHC affinity influences TRM formation and integrates with additional local signals to convert effector CD8^+^ T cells into long-lived residents. By positioning TRMs as pivotal mediators of local immunity, we highlight potential strategies to modulate TRM-inducing signals—bridging mechanistic insights from infection and cancer toward translational applications.

## 2 Lessons learned from the classic subpopulations of memory T cells

**Figure 1:**
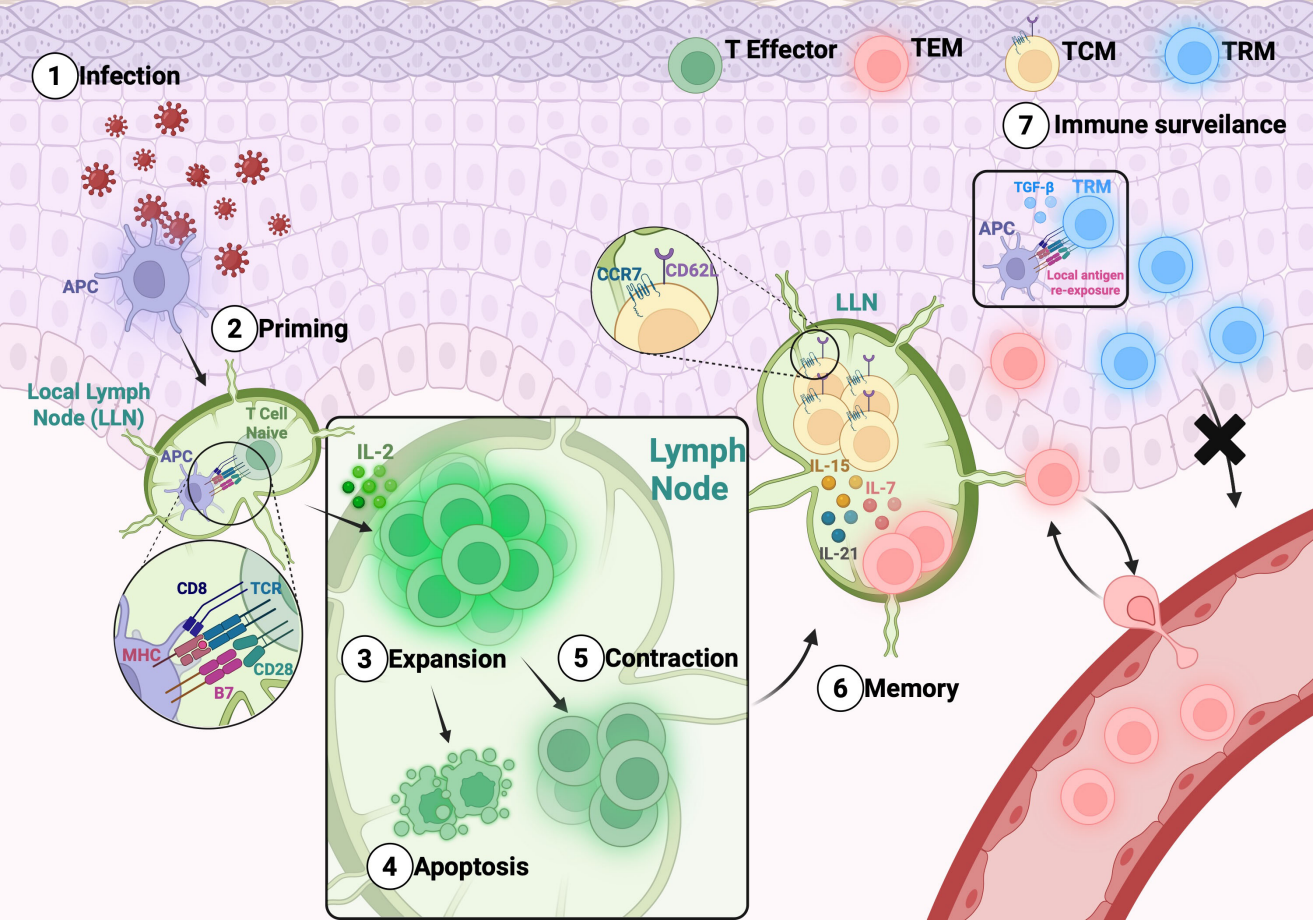
General overview of the memory T cell subsets generation and maintenance:. After infection (1), the differentiation of memory T cells includes a priming phase (2) in the LN. After expansion (3) the antigen is cleared, and part of the T cells enter in apoptosis (4) and part survive – contraction phase (5). Further, T cells differentiate into TCM, which circulate between the blood and secondary lymphoid tissues, and TEM, which exit the LN to enter the circulation and peripheral tissues (6). TRM cells are generated from T effector differentiation that migrate to the tissue and remain there to rapidly face antigen challenge. TRMs can recognize antigens within tissues and, upon stimulation with cytokines such as TGF-β, can induce or enhance the expression of residency markers (7).

Memory T cells are essential to long-term protection against infectious diseases and contributes for antitumor immunity. Their generation and maintenance have been extensively investigated, particularly in the settings of viral infections and cancer (19–21). Upon recognition of antigens presented by Major Histocompatibility Complex class I (MHC I) molecules on Antigen-Presenting Cells (APCs), along with co-stimulatory and cytokine signals, naïve CD8^+^ T cells become activated and undergo clonal expansion and differentiate into effector cells that produce IL-2 and mediate pathogen clearance. Following antigen elimination, most of these cells, approximately 90%, undergo apoptosis during the contraction phase, while a minority survive and initiate the memory differentiation program. This process gives rise to two major subsets: short-lived effector cells (SLECs) and memory precursor effector cells (MPECs) (22). The developmental bifurcation between these subsets is governed by distinct transcriptional and signaling programs. MPECs, which eventually give rise to long-lived memory T cells, integrate multiple cues such as weaker TCR stimulation (23), cytokines like IL-15 (24), and transcriptional regulators including Bcl-6 (25) and Eomes (26), in addition to epigenetic mechanisms that stabilize the memory gene expression profile (27). The transcription factors Forkhead box protein O1 [FoxO1] (28) and T cell factor 1 [Tcf-1] (29) are central to this process: both are highly expressed in naïve CD8^+^ T cells, transiently downregulated during effector differentiation, and subsequently re-expressed as cells transition toward the memory state. This dynamic regulation indicates that the transcriptional circuitry underlying memory formation is preconfigured in naïve cells, temporarily suppressed during effector commitment, and reactivated to sustain longevity and homeostatic responsiveness. In contrast, SLECs fail to reinitiate this memory-associated program, resulting in terminal differentiation and apoptosis (30). Consequently, only a small fraction—approximately 5%—of the activated CD8^+^ T cell population persists as long-lived memory cells (31). A schematic overview of the memory T cell subsets generation and maintenance is present in **Figure 1**.

The maintenance and differentiation of TEM and TCM cells rely heavily on the expression of cytokine receptors such as IL-7, IL-15, and IL-21, features that have been extensively characterized (32, 33) which dependent on the expression of CD122 (IL-2/IL-15 receptor β chain) and CD132 (common gamma chain, γc) (34, 35).Traditionally, memory T cell subsets have been classified based on their tissue localization, surface marker expression, and functional attributes. Following differentiation, TCMs predominantly reside in lymphoid tissues, whereas TEM cells circulate through the blood, secondary lymphoid organs, and peripheral tissues. TCMs are marked by the expression of lymph node homing receptors CD62L and CCR7, which are absent in TEM cells (36, 37).

Following antigen clearance, memory T cells initiate a developmental program that supports continued clonal expansion. Remarkably, these cells can undergo up to eight rounds of division in the absence of cognate antigen, a process sustained by homeostatic signals from γc receptor cytokines (38–40). Compared to their naïve counterparts, memory T cells possess lower activation thresholds, requiring reduced antigen levels and shorter TCR signaling durations to become fully activated (41). Transcription factors (TF) play a central role in the rapid recall capacity of memory CD4^+^ T cells. While T-bet is indispensable for IFN-γ expression in naïve T cells, memory CD4^+^ T cells can rapidly produce this cytokine without upregulating T-bet. Instead, this response is linked to enhanced nuclear translocation of NFκB p50, suggesting that increased accessibility of promoter regions allows more efficient NFκB recruitment, thereby driving accelerated gene expression during recall responses (42). The molecular mechanisms governing the induction and maintenance of T cell memory have been thoroughly reviewed elsewhere (43–46).

TRM cells arise within a signaling framework partially shared with other memory T cell subsets but acquire unique features due to their differentiation and function across diverse tissues. Their development is shaped by local environmental cues, including variations in antigen availability, differential expression of adhesion molecules, distinct cytokine and chemokine responsiveness, and tissue-specific transcriptional programs. Consequently, the biology of TRMs diverges substantially from that of other memory T cell subsets, and their presence is strongly associated with immune protection. These characteristics critically influence T cell responses in both viral infections and cancer.

## 3 What drives a memory CD8^+^T cell to become a TRM?

TRM cell differentiation follows a distinct pathway compared to other memory T cell subsets. Evidence indicates that CD8^+^ effector T cells, once primed in local lymph nodes, acquire TRM lineage traits and subsequently migrate to peripheral tissues through the bloodstream (13). As the infectious process begins to resolve, a subset of these pre-committed effector cells persists at the site of inflammation and differentiates into CD8^+^ TRM cells under the influence of local environmental cues, including homing molecules, inflammatory cytokines, and chemokines (47, 48). For instance, during viral infections, CD8^+^ T cells expressing P-selectin ligands migrate along CXCL10 gradients produced by endothelial cells in response to type I interferons (49). Like TEM and TCM, it is now understood that primary TCR signaling in the lymph node (50) together with local inflammatory signals, cytokines, and chemokines, plays a sequential and collaborative role in the generation, proliferation, and maintenance of TRMs (51). However, the precise identity and timing of these signals vary across tissues and remain incompletely understood. The following section explores the molecular and environmental factors that guide effector CD8^+^ T cells toward tissue-specific TRM differentiation.

### 3.1 The influence of TCR signaling to form TRMs

TCR signaling plays a crucial role in inducing the expression of adhesion molecules characteristic of TRMs. In a mouse model of intranasal viral-vectored immunization using a *Mycobacterium tuberculosis* antigen, antigen-specific CD8^+^ T cells began expressing CD103 shortly after entering lung tissue, and its expression increased during the contraction phase (52). A similar pattern was observed in mice infected intranasally with influenza virus, where effector CD8^+^ T cells recognizing their cognate antigen in the lung upregulated CD103 and maintained CD69 expression (53). Consistent findings in intestinal (54) and skin (50) models confirm that local antigen recognition is essential for efficient TRM formation. In a mouse kidney transplant model, the persistence of antigens presented by recipient-derived dendritic cells was also crucial for maintaining TRM stability and function (55). Likewise, in viral infection models, antigen availability determined TRM differentiation and longevity. For instance, intracerebral delivery of vesicular stomatitis virus (VSV) antigens was required to sustain CD69 and CD103 expression on brain CD8^+^ T cells, with CD103^+^ clusters persisting up to 30 days post-infection, even after viral clearance (56).

Although cognate antigen is essential for TRM formation, the recruitment of antigen-specific circulating effector T cells into tissues can also be driven by local inflammatory cues. In this context, tissue-derived cytokines and chemokines promote TRM differentiation (48). This was demonstrated in a skin infection model with HSV, where inflammation induced solely by the contact sensitizer 2,4-dinitrofluorobenzene (DNFB) was sufficient to recruit previously generated antigen-specific CD8^+^ T cells, which subsequently expressed CD103 and differentiated into TRMs (57). In the same model, broader tissue protection mediated by TRMs was achieved through localized antigenic restimulation. Repeated antigen exposures in distinct skin regions generated spatially dispersed TRMs, whereas a single high-dose injection at one site led to TRM formation restricted to that specific area (58). Conversely, the generation of pulmonary CD8^+^ TRMs, as well as the differentiation of circulating effector T cells, critically depends on local antigen recognition within the lung. Thus, although inflammatory cues such as cytokines and chemokines can recruit circulating effector CD8^+^ T cells to the pulmonary tissue, these signals alone are insufficient to induce TRM differentiation. Only upon cognate antigen encounter *in situ* do these effectors cells acquire the transcriptional and phenotypic features characteristic of long-lived TRMs.

The affinity between the MHC I–peptide complex and the T cell receptor is a key determinant guiding effector T cells toward the TRM phenotype. Frost et al. (59) demonstrated this relationship by isolating memory cells from the spleen, brain, and kidney of mice intracerebrally infected with polyomavirus, a virus that preferentially replicates in the kidney. Upon ex vivo antigen challenge, TRMs from the brain and kidney displayed approximately 20-fold higher peptide affinity compared to splenic memory cells, suggesting that TRMs originate from high-affinity effector cells that infiltrate non-lymphoid tissues during acute infection. This finding implies that TRMs may possess distinct TCR repertoires compared with other antigen-specific memory subsets. Conversely, in a systemic polyomavirus infection model, higher numbers of brain CD8^+^ TRMs were observed in groups primed with subdominant epitopes that generated weaker TCR stimulation (60). Likewise, in a murine influenza model, CD8^+^ T cells with lower TCR affinity exhibited a competitive advantage in forming lung TRMs, although affinity did not appear to influence their persistence or functional quality (61). Finally, in a study using adoptive transfer of transgenic CD8^+^ T cells specific for lymphocytic choriomeningitis virus (LCMV), followed by ear skin infection with Vaccinia virus expressing the LCMV peptide GP33, the presence of cognate antigen was required for IFN-γ production and TRM formation. When Vaccinia variants expressing a modified SIINFEKL peptide with reduced affinity for the OT-I TCR were used, the strength of TCR engagement was shown to modulate both IFN-γ expression and the transcriptional programs underlying TRM differentiation (62).

The contribution of cognate antigen to TRM-mediated protection has been demonstrated across several models, including malaria vaccination targeting the liver (63, 64) hepatitis C mouse models (65), and the female reproductive tract during genital HSV-2 infection (66). Together, these studies reinforce that antigen presentation and the strength of TCR engagement are central to CD8^+^ T cell differentiation into TRMs. Nevertheless, the precise influence of peptide-MHC: TCR affinity on TRM generation and tissue establishment remains incompletely defined and appears to vary according to factors such as infection type and immunization route. **Table 1** summarizes representative studies highlighting this variability in TCR interaction strength and its relationship with TRM formation.

**Table 1:** Studies on peptide–MHC: TCR affinity in the format ion of CD8 TRMs.

Model/Tissue	How affinity/Signal was measured or manipulated	Main finding	Reference
Mouse — circulating CD8^+^ memory (not specific to TRM)	Genetic/pharmacologic reduction of TCR signaling and antigen affinity variation	Lower signaling/affinity can favor memory — reduced TCR signaling accelerated and increased CD8^+^ memory generation.	(23)
Mouse — brain & kidney (Mouse polyomavirus infection)	2D affinity (micropipette), tetramer binding	High affinity favored — TRM displayed ~20× higher TCR affinity than splenic memory cells.	(61)
Mouse — brain (Polyoma virus infection)	Varied antigenic stimulation strength	Lower affinity favored/better function — suboptimal stimulation recruited more functional brain TRM; strong TCR signals impaired memory development.	(62)
Mouse — lung (Influenza A infection)	OT-I epitope variants with high vs low TCR affinity	Lower/intermediate affinity favored— pulmonary TRM formation was higher with weaker TCR stimulation; strong signals reduced TRM generation.	(63)
Mouse — skin/ear (Vaccina virus model)	Manipulated signal intensity; readout: Blimp1, CXCR6, S1PR1	Mixed/context dependent. Stronger signaling promoted CXCR6^+^ retention and inhibited egress via S1PR1; both high and moderate signals contributed differently.	(65)
Mouse — brain (chronic infection with Toxoplasma gondii)	Comparison among T cell clones of different TCR affinities	High affinity favored — higher affinity clones trafficked better to the brain and generated more CD103^+^ TRM.	(67)
Review — lung/trachea (human and mouse)	Compilation of multiple experimental studies	Contextual/heterogeneous — affinity/signal impact varies by tissue and microenvironment; in lungs, lower affinity can sometimes favor TRM.	(68)
Review — respiratory tract	Review of multiple models and affinity assessments	Context-dependent — lung microenvironment and inflammation modulate how TCR affinity/signal strength shapes TRM formation.	(69)

Mouse polyoma virus infection (61), Toxoplasma gondii infection (67), Vaccinia virus model (65), Lung Influenza infection (63), Mouse polyomavirus infection in brain (62), Listeria monocytogenes strain carrying ovalbumin peptides (23), Review in lung TRMs (68), TRM in respiratory virus infection (69).

### 3.2 The expression of functional TRMs markers for tissue lodging and location

Beyond antigen-driven TCR signaling, CD8^+^ TRM lymphocytes depend on specific molecules that support their long-term residence within tissues (70). Upon entry, pre-committed effector CD8^+^ T cells activate transcriptional programs that consolidate the TRM phenotype, notably through the expression of adhesion and retention molecules (71, 72). Among these, CD69, CD103, and CD49 have been widely used as defining markers, although their functional relevance varies across tissues and microenvironmental contexts (73–75).

CD69 is strongly upregulated upon TCR-mediated lymphocyte activation (76) and plays a key role in tissue retention, metabolism, and maintaining an activated T cell phenotype (77). Mechanistically, CD69 binds in cis to sphingosine-1-phosphate receptor 1 (S1P1R) on the T cell membrane, triggering receptor internalization and degradation (78). Without S1P1R, T cells cannot respond to circulating sphingosine-1-phosphate (S1P), resulting in their retention within tissues (15, 79). Through this mechanism, early CD69 expression effectively inhibits T cell egress and promote TRM residency.

CD49a (very late antigen 1, VLA-1; α1β1 integrin) binds collagen IV in non-lymphoid tissues (80, 81). Its expression occurs early during CD8^+^ TRM generation. For instance, following mouse intranasal immunization with a viral-vectored *M. tuberculosis* antigen, most antigen-specific CD8^+^ T cells arrived in the lung already expressing CD49a, with expression enriched during contraction and memory phases (52). Comparable early CD49a expression was noted in a skin HSV-1 infection model. Interestingly, in both studies, CD49a was shown not to be essential for the generation of CD8^+^ TRM lymphocytes in the tissue, as these cells arrive already expressing this marker. Although CD49a is not required for TRM generation, it correlates with IFN-γ production by skin TRMs during HSV infection and contributes to their maintenance and effector function within tissues (82).

CD103 (αEβ7 integrin) is a hallmark marker expressed on CD8^+^ TRMs in infected tissues and certain tumors (83, 84). This integrin binds to E-cadherin, which is expressed on epithelial cells (85). In human tumors such as glioblastoma and breast cancer, elevated CD103 expression correlates with better prognosis, likely due to enhanced CD8^+^ TRM infiltrations (86–88). CD103 expression on effector CD8^+^ T cells is regulated by TGF-β. In viral infections, TGF-β promotes CD103 upregulation during the contraction phase in skin and lungs, aiding TRM differentiation in mouse (55, 89). Similarly, studies in a lung tumor (90) and HPV tumor models (91) have also shown that TGF-β induces the upregulation of CD103 on the surface of CD8^+^ T lymphocytes, contributing to the formation of TRMs. CD103 is regarded as a reliable molecular marker for the characterization of CD8^+^ TRMs, particularly those residing in epithelial tissues. Together with CD103, the identification of TRMs is often further supported by the co-expression of CD69 and CD49a (70).

Despite frequent use of CD69, CD103, and CD49a to identify CD8^+^ TRMs, their expression is neither universal nor uniform, varying with tissue environment and inflammation. For example, CD69 is essential for TRM formation in the kidney, where its enforced expression promotes residency, but dispensable in the small intestine, illustrating tissue-specific requirements (92). Similarly, CD103 and CD49a expression depend on local cytokines and cell interactions such as TGF-β and integrin signaling and may be absent in certain tissues or disease contexts (93). These findings highlight TRM phenotypic heterogeneity and underscore the importance of tissue and disease-specific considerations in TRM characterization.

Altogether, integrin’s receptors seem to act in concert to lodge CD8^+^ T cells that are becoming TRM cells in their respective tissue - be it in lung, kidney (94), brain (95), intestine (96), skin (97), and female reproductive tract (98). However, we still know relatively little about the identity of site-specific molecules that guide TRM cells to reside in one tissue over another. Despite growing evidence of tissue-specific modulators influencing TRM marker expression, the precise molecules and mechanisms governing site-selective TRM localization remain poorly defined, warranting further investigation.

### 3.3 The role of cytokines in controlling TRM dynamics

Early studies on mouse memory CD8^+^ T lymphocytes suggested that their long-term maintenance depends less on continuous TCR stimulation and more on homeostatic signals (99–101). This paradigm is similarly observed for CD8^+^ TRMs, which appear to rely minimally on persistent antigen exposure for their maintenance. However, the homeostatic mechanisms governing TRMs may diverge from those regulating other memory subsets. Unlike lymphoid organs, barrier tissues are frequently exposed to environmental antigens and inflammatory stimuli, which generate unique local cues that support TRM survival and persistence.

Among these tissue-specific factors, TGF-β has emerged as a pivotal cytokine orchestrating the differentiation, retention, and long-term maintenance of CD8^+^ TRMs in epithelial barrier tissues by inducing key retention molecules such as CD103 and CD69 that inhibit lymphocyte egress (102, 103). In LCMV infection models, effector CD8^+^ T cells migrate to the intestinal mucosa where TGF-β drives CD103 expression independently of ongoing antigen stimulation, highlighting its essential role in imprinting tissue residency post-pathogen clearance. Furthermore, ex vivo stimulation of splenic CD8^+^ T cells from LCMV-infected mice with TGF-β combined with IL-33 or TNFα enhances the expression of TRM-associated markers including CD69 and Ly6C, reinforcing the interplay between cytokines in TRM programming (104). Importantly, TGF-β’s role extends beyond the intestine, controlling CD8^+^ TRM maintenance in diverse tissues such as skin (89), kidney (105) and genital tract (91) in mouse models, indicating a conserved mechanism by which TGF-β integrates environmental signals to establish a stable TRM phenotype that ensures effective localized immune surveillance.

Interleukin-15 (IL-15) signaling via its receptor (IL-15R) is essential for the survival and homeostatic proliferation of CD8^+^ TRMs, enabling their long-term persistence similarly to TEM cells (13, 89). Antigen-specific CD8^+^ TRMs in tissues such as liver, kidney, skin, and salivary glands actively proliferate in response to IL-15 stimulation (106). In contrast, TRMs in certain sites like the small intestine, pancreas, and female reproductive tract show a reduced dependence on IL-15 despite retaining responsiveness, suggesting that local cytokine milieu distinctly influences TRM homeostasis (107). The balance between IL-15 and interleukin-7 (IL-7) signaling further fine-tunes TRM adaptation, notably, high levels of both cytokines lead to downregulation of IL-7 receptor alpha (IL-7Rα), whereas IL-7Rα is upregulated when IL-15 is scarce (108). This reciprocal regulation exemplifies a sophisticated homeostatic network enabling TRMs to tailor their survival strategies according to tissue-specific cytokine availability. Overall, IL-15 functions as a central, yet context-dependent, regulator of CD8^+^ TRM persistence and self-renewal across non-lymphoid tissues.

Beyond IL-15 and IL-7, additional cytokines contribute to TRM maintenance and function, often in a tissue-specific manner. For example, in murine models of pulmonary infection, alveolar macrophages cross-present antigens to CD8^+^ T cells while producing IL-18, which promotes lung TRM differentiation. In this context, mice deficient in the IL-18 receptor display impaired induction of CD103^+^ antigen-specific CD8^+^ T cells in the lung, suggesting that IL-18 indirectly supports TRM development by promoting CD4^+^ Th1 cell differentiation and shaping the local cytokine milieu (74). Similarly, IL-21 partially regulates the differentiation of intestinal CD8^+^ effector T cells into TRMs, as shown in mixed bone marrow chimera models containing IL-21 receptor-deficient and wild-type cells (109). In the brain, increased IL-21 receptor expression on CD8^+^ T cells during murine polyomavirus infection correlates with higher CD103 levels and enhanced TRM formation (110). Additionally, IL-33 has emerged as another cytokine with a crucial role in sustaining intestinal CD8^+^ TRMs, although the precise mechanisms remain under investigation (111).

In summary, cytokines are fundamental regulators of CD8^+^ TRM dynamics, influencing their differentiation, maintenance, and functional adaptation within diverse tissue microenvironments. Key cytokines such as TGF-β and IL-15 coordinate a delicate balance between tissue retention, homeostatic proliferation, and survival, while others, including IL-7, IL-18, IL-21, and IL-33, modulate TRM biology in a tissue-specific manner. Elucidating these cytokine-driven pathways will be critical for the development of targeted immunotherapies and vaccination strategies aimed at harnessing or enhancing tissue-resident immune memory.

### 3.4 Chemokines coordinate the migration and tissue distribution of TRMs

While cytokines are central to the differentiation and maintenance of CD8^+^ TRMs, chemokines and their receptors play an equally critical role in shaping the tissue-specific localization, migratory behavior, and functional adaptation of these cells. Chemokine signaling is essential not only for recruiting effector T cells to non-lymphoid tissues but also for orchestrating their retention, positioning, and morphological transformation into TRMs.

The skin provides a well-characterized model of how chemokine-mediated cues drive TRM development and function. As a barrier tissue with constant environmental exposure, it establishes a specialized microenvironment where TRMs intercalate among keratinocytes and adopt a dendritic-like shape that optimizes local immune surveillance (112). The expression of chemokine receptor CXCR6 is required for CD8^+^ T cells to differentiate into TRMs and acquire this morphology, facilitating their integration among keratinocytes (113). Among these, the CXCL16–CXCR6 interaction stands out as a key axis promoting epidermal TRM retention and survival (101). In parallel, CCR10 and its ligand CCL27, produced by keratinocytes, mediate skin-specific homing of T cells (114). Collectively, these chemokine pathways ensure proper TRM localization and persistence, supporting rapid and effective cutaneous immune protection.

Beyond tissue retention, chemokines play a pivotal role in guiding the initial migration of effector CD8^+^ T cells from the circulation into peripheral tissues (115). Once within the tissue, the pre-committed effector cells can differentiate into TRMs, even in the absence of cognate antigen recognition, a process known as local heterotypic differentiation (116). One of the earliest detailed demonstrations of this mechanism arose from studies of viral infection in the female reproductive tract. Upon secondary antigen encounter, resident CD8^+^ TRM cells rapidly produced interferon-gamma (IFN-γ), along with the chemokines CXCL9 and CXCL10, which acted on local endothelial cells to modify the vasculature and facilitate the recruitment of additional memory T cells from the circulation. These newly recruited cells, drawn into the inflamed tissue microenvironment, subsequently differentiated into TRMs and expanded the local pool of tissue-resident memory cells (117). This dynamic indicates that the exclusion of memory T cells from certain non-lymphoid compartments is not absolute, but rather context-dependent, and can be transiently overridden when local TRMs initiate a chemokine-driven “alarm” in response to antigen recognition. Such mechanisms likely serve to accelerate the recruitment of pathogen-experienced T cells to sites of reinfection, thereby enhancing immune responsiveness. Supporting this concept, additional studies in human skin have shown that expression of the chemokine receptor CCR8 on CD8^+^ T cells correlate with the expression of canonical TRM markers, including CD103 and CD69, as well as with gene expression profiles associated with tissue residency (118). These findings underscore the close link between chemokine receptor expression, migratory cues, and the establishment of the resident memory phenotype in peripheral tissues.

In the central nervous system (CNS), chemokine-driven recruitment of CD8^+^ T cells are often associated with inflammation or tissue damage rather than protective immunity. During aging, the basal release of chemokines—including CCL5, CCL11, CXCL9, and CXCL10 promotes the infiltration of CD8^+^ T cells into the CNS. This process appears to be largely antigen-independent and stochastic, often associated with ischemic injury. Once inside the CNS, these infiltrating cells can give rise to a population of “age-associated” CD8^+^ TRMs characterized by the expression of CD103, CD69, and PD-1 (119). In contrast, in mucosal sites chemokine signaling can also sustain protective TRM responses. In the genital mucosa, for instance, HSV-1 infection does not always lead to lesion formation; instead, resistance correlates with local CXCL17 expression and the recruitment of CD8^+^ T cells bearing its receptor, CXCR8. These CD8^+^CXCR8^+^ cells acquire a TRM phenotype and confer enhanced protection against recurrent herpes lesions (120).

**Figure 2:**
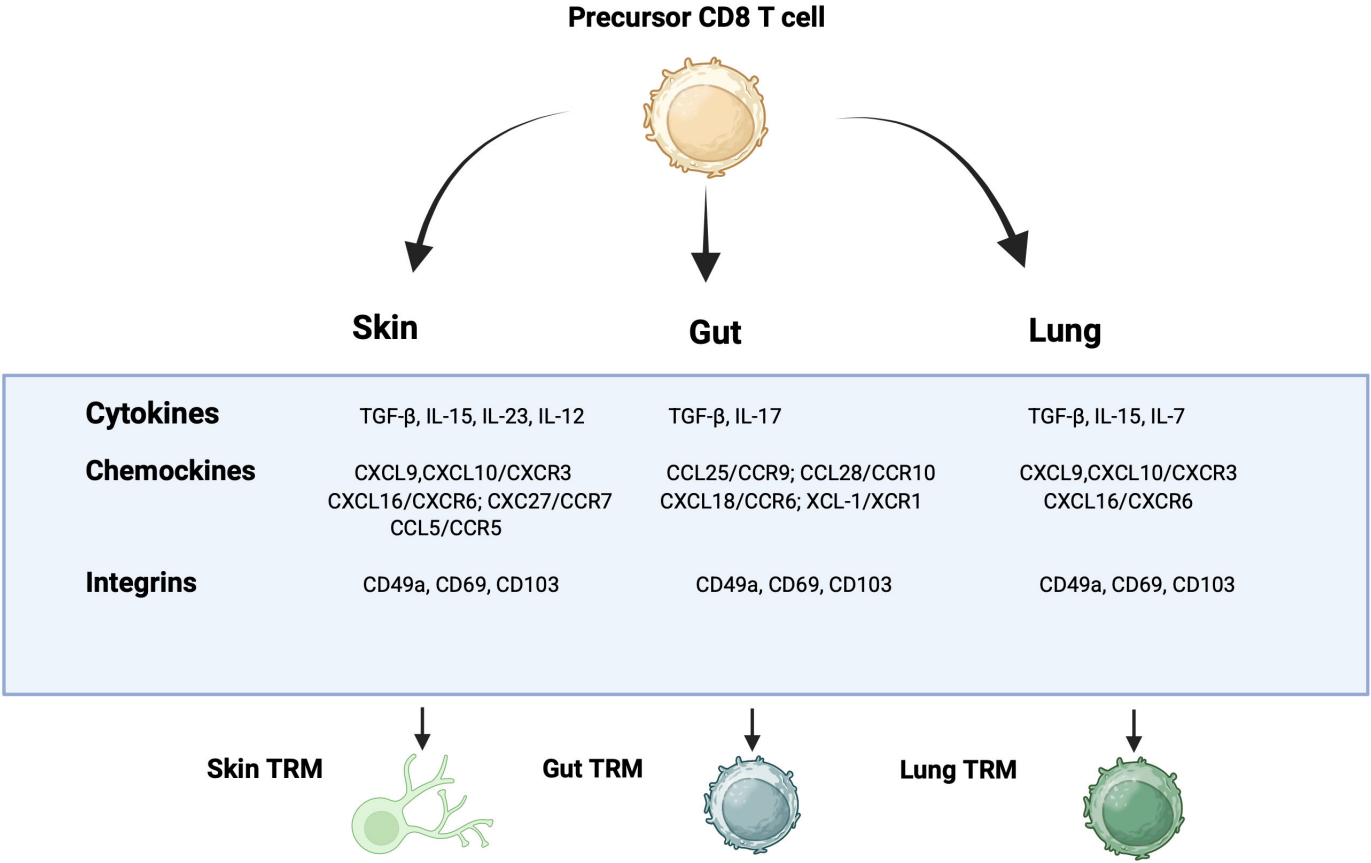
Cytokines, chemokines, and integrins involved in the differentiation of CD8 effector T cells into CD8 TRMs in the skin, gut, and lung.

Compared to canonical TRM markers CD103 and CD69, the expression of chemokines and their receptors is more tightly associated with the anatomical localization and functional specialization of TRMs. Some chemokine receptors primarily guide the localization of TRMs within tissues, while others directly influence their activation, morphology, or survival. For instance, in the salivary glands, sites of cytomegalovirus infection and replication, CXCR3-mediated CD8^+^ T cell migration appears to be an intrinsic property of the tissue rather than a response to infection. The salivary gland constitutively recruits CD8^+^ T cells in a CXCR3-dependent manner and is capable of inducing and sustaining TRM populations even in the absence of infection, antigen, or inflammation (121). Moreover, the CXCR3–CXCL10 signaling axis is essential for TRM recruitment and accumulation (122), while the CXCL16–CXCR6 pathway has been shown to promote the retention of CD8^+^ TRMs in barrier tissues such as the skin and lungs (123, 124). Collectively, these findings highlight that chemokines function not merely as passive recruiters of T cells but as active regulators in shaping TRM functions in the tissues. They mediate the initial recruitment of effector cells, modulate their retention, influence the expression of residency-associated markers, and tailor TRM functionality according to the specific demands of the local tissue microenvironment. As such, chemokines are indispensable components of the tissue residency program, acting in concert with cytokines and other local signals to establish robust, site-specific immune memory. In **Figure 2**, we illustrate the principal cytokines, chemokines, and integrins involved in CD8^+^ TRM formation and maintenance across the most studied tissues (skin, gut, and lung).

### 3.5 Signals for TRM differentiation are driven by the kinetics of transcription factors in tissues

CD8^+^ TRM cells exhibit tissue-specific transcriptional programs that are largely established during the peak of the effector T cell response (125, 126). Upon activation within non-lymphoid tissues, effector CD8^+^ T cells initiate a transcriptional cascade that programs them toward a resident memory phenotype (13, 127). For example, in the skin, only effector cells that activate the appropriate gene expression profile and receptor repertoire adopt a dendritic morphology and become functional TRM cells (113). The transcription factor Krüppel-like factor 2 (KLF-2) promotes the expression of S1PR1, enabling tissue egress, but is downregulated after TCR activation. While IL-15 induces KLF-2, TNF-α, IL-33, and TGF-β cooperate to suppress its expression (128).

A central component of TRM differentiation is the modulation of T-box transcription factors, particularly T-bet and Eomesodermin (Eomes). Upon antigen encounter, activated CD8^+^ T cells produce IL-2, which induces Eomes expression (129). Eomes is associated with the promotion of long-lived memory cells by enhancing self-renewal and proliferative potential (130). In contrast, T-bet is predominantly expressed in early effector CD8^+^ T cells, where it supports cytotoxic function and inflammatory responses. As T cells transition to memory states, both T-bet and Eomes levels decline (131, 132). A low T-bet/Eomes expression ratio favors memory formation, and further downregulation of both factors is required for the acquisition of tissue residency. In the lungs, TRM cells expressing high levels of CD103 and CD69 exhibit notably suppressed T-bet levels (133). Similarly, in the skin, the downregulation of both Eomes and T-bet is critical for the infiltration, retention, and survival of TRM cells in the epidermis. Also, residual T-bet expression maintains CD122 expression and IL-15 responsiveness, supporting TRM survival in skin (89). Conversely, the expression of T-bet and Eomes is associated with CCR7, a receptor that promotes lymphoid tissue recirculation and favors the differentiation of CD8^+^ T cells into TEM or TCM subsets (134). Thus, the coordinated repression of T-bet and Eomes can be one of the hallmarks of TRM differentiation, facilitating tissue adaptation and the quiescent phenotype necessary for long-term residency, particularly in epithelial barriers.

Runt-related transcription factor 3 (Runx3) is a transcription factor involved in cellular development, inflammatory responses, and tumor suppression (135, 136). In the context of TRM biology, Runx3 has been identified as an essential regulator of lineage commitment and homeostasis (137). It promotes the expression of canonical TRM markers while repressing genes linked to tissue egress, including S1pr1 (128). Functional studies in murine models of LCMV infection and melanoma demonstrated that deletion of Runx3 results in a dramatic reduction, ranging from 50- to 150-fold in CD8^+^ T cells expressing CD69 and CD103 across various tissues, including salivary glands, kidneys, skin, and lungs (138). Notably, Runx3 appears to function in a tissue-independent manner, reinforcing its role as a core component of the TRM transcriptional program. Runx3 regulates a transcriptional network that controls T cell migration and effector functions within non-lymphoid tissues (139). A deeper understanding of how Runx3 governs TRM differentiation may prove critical for advancing tissue-targeted immunotherapies that harness CD8^+^ TRMs.

Additional transcription factors also contribute to TRM development in a tissue-specific manner. After effector CD8^+^ T cells infiltrate tissues such as the skin, small intestine, liver, and kidneys, the expression of Hobit and Blimp-1 becomes essential for further differentiation. These factors promote TRM formation by repressing genes involved in circulation, such as CCR7 and S1PR1. Although Blimp-1 is not strictly essential, it cooperates with Hobit to drive the expression of TRM-associated surface markers including CD49a, CD69, and CD103 (126, 140). During pulmonary influenza infection in mice, Blimp-1 expression in terminal effector CD8^+^ T cells favor the development of TRMs over TCMs. In this model, once effector CD8^+^ T cells reach the lungs, Blimp-1 suppresses the expression of T cell factor-1 (TCF-1) in CD8^+^ TRM cells, a mechanism that promotes TRM differentiation over TCM, as TCF-1 is essential for the development of the TCM lineage. This transcriptional program may facilitate the conversion of circulating CD8^+^ T lymphocytes into tissue-resident cells, with Blimp-1 acting as a key regulator by antagonizing the TCM differentiation pathway (126). In the context of chronic viral infection, as shown in LCMV models, Hobit expression is confined to the TRM lineage, whereas Blimp-1 is broadly expressed across all CD8^+^ effector and memory subsets (141). These findings suggest that Hobit is a more specific marker of TRM identity, while Blimp-1 serves a broader role in memory CD8^+^ T cell differentiation by inhibiting TCF-1 and promoting TRM commitment.

**Figure 3:**
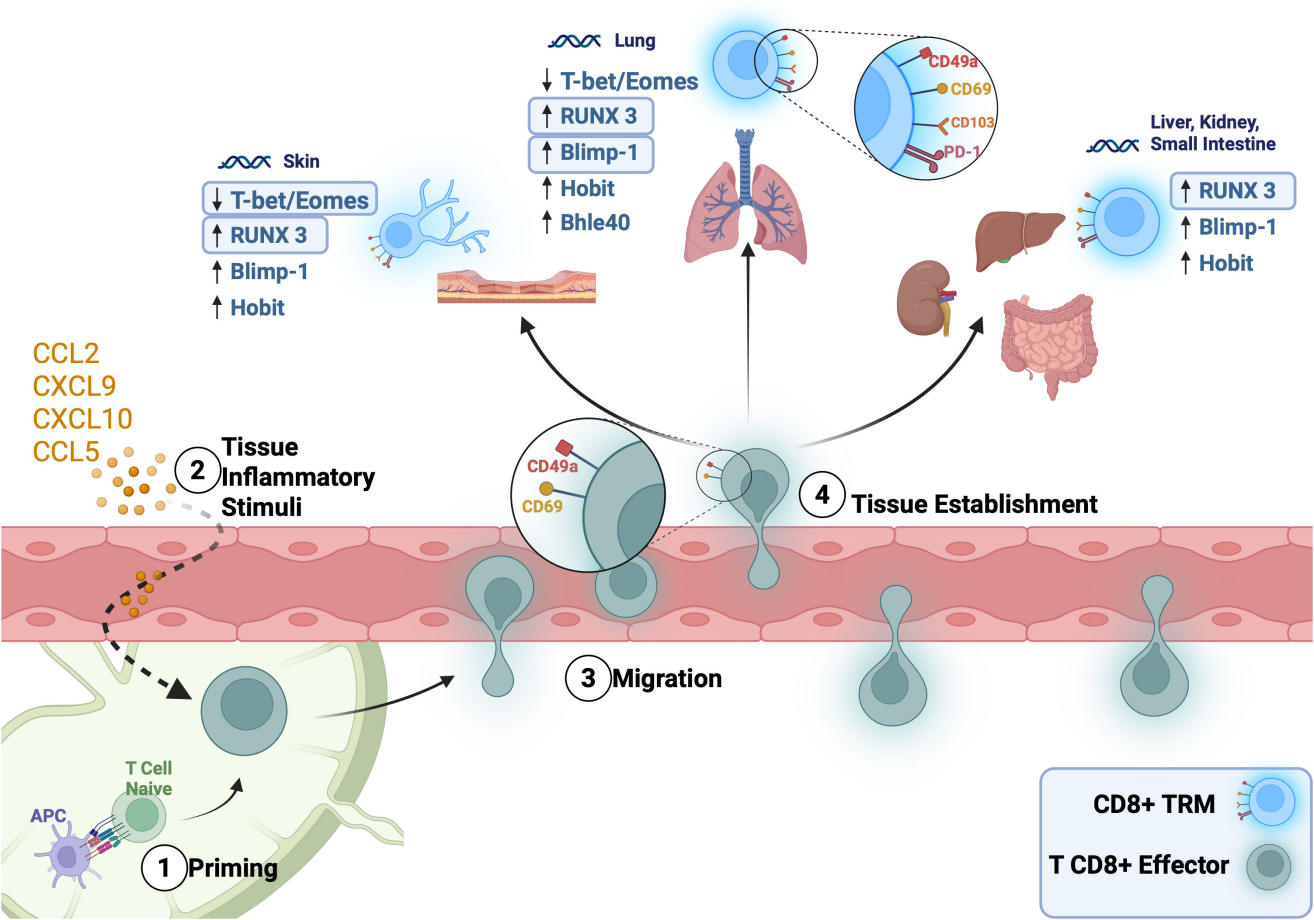
Migration and formation of CD8^+^ TRMs in different tissues in the context of infection or injury and the expression of specific transcriptional factors. After being primed in the regional lymph nodes (1), and in response to inflammatory stimuli from the tissue that produces chemokines (2), effector CD8 T lymphocytes follow these signals and migrate into the inflamed tissue, expressing CD69 and CD49a (3). In this microenvironment, CD8+ T lymphocytes that express CD69 downregulate S1PR1, which leads to their retention in the tissue (4). Within each specific tissue, CD8 T lymphocytes respond to TGF-β and other local microenvironmental cues that induce transcription factors, promoting the expression of CD49a and other canonical functional markers of TRMs, such as CD103 and PD-1.

Another key factor, the basic helix-loop-helix family member e40 (Bhlhe40), has recently gained attention for its role in sustaining CD8^+^ TRM cell metabolism and function, particularly under inflammatory conditions. Initially studied in pulmonary TRMs, where it supports mitochondrial function and cytokine production (e.g., IFN-γ) (142). Bhlhe40 has also been shown to be essential for TRM maintenance in other tissues. In the brain, Bhlhe40-deficient CD8^+^ T cells exhibit impaired persistence and effector function following viral infection. In the small intestine, Bhlhe40 limits T cell exhaustion and sustains TRM metabolic fitness during chronic infection (143). In the liver, it contributes to the regulation of intrahepatic TRM homeostasis and modulates inflammatory potential during hepatotropic viral infections (144). Moreover, Bhlhe40 expression is suppressed by PD-1 signaling, and its regulation has been implicated in the responsiveness of TRMs to anti-PD-1 immunotherapy (145). Thus, some transcription factors involved in TRM formation are common across most tissues, while others are more tissue-specific, as shown in **Figure 3**.

**Figure 4:**
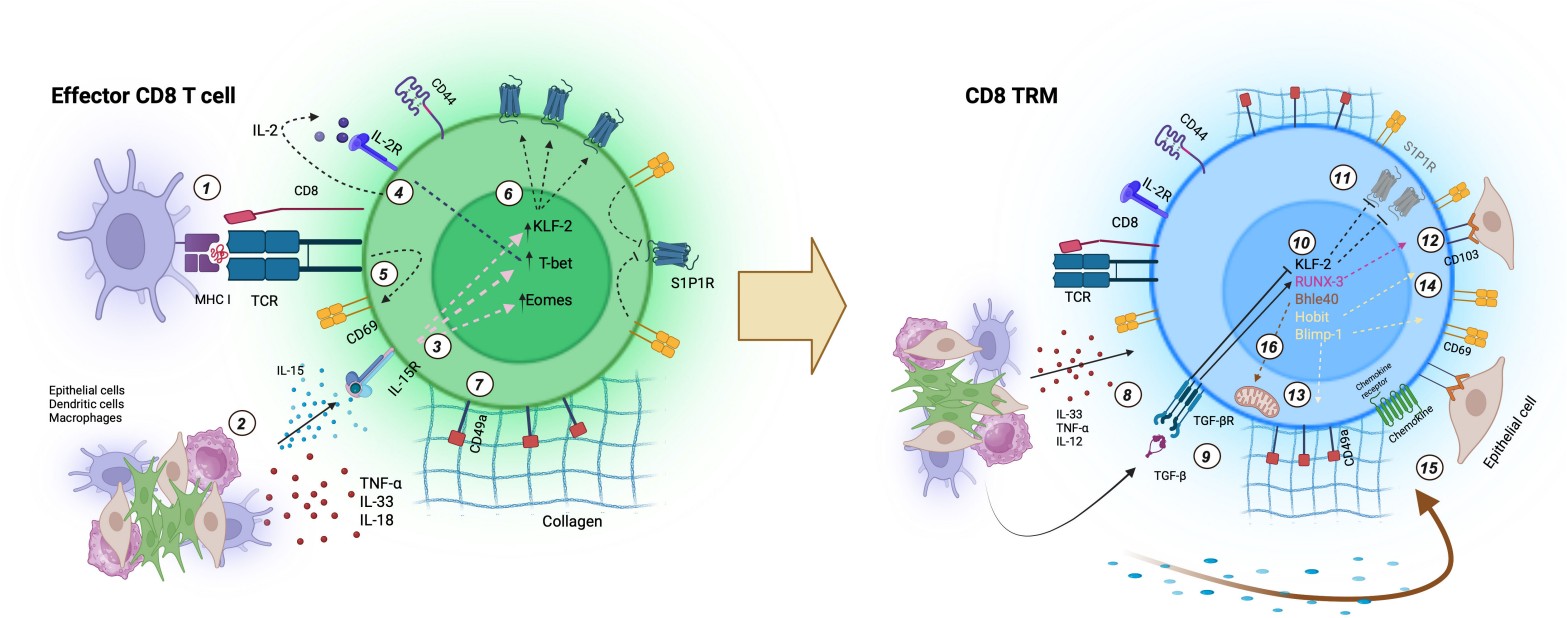
Integrated model of signals driving CD8^+^ TRM differentiation: 1) TCR engagement with peptide–MHC I complexes (low or high affinity) provides activation signals; 2) Local cues derived from pathogens, injury, and inflammation—including TNF-α, IL-18, IL-33, and IL-15—further shape TRM differentiation; 3) IL-15 trans-presentation induces the transcription factors KLF2 and T-bet/Eomes; 4) Autocrine IL-2 reinforces T-bet expression; 5) TCR activation upregulates CD69 expression; 6) KLF2 promotes S1PR1 expression, regulating tissue egress; 7) Effector CD8^+^ T cells begin expressing CD49a; 8) Production of TNF-α, IL-18, and IL-33 is maintained or amplified locally; 9) Epithelial cells, macrophages, and dendritic cells produce TGF-β, which binds to TGF-β receptors on CD8^+^ T cells. 10) TGF-β signaling downregulates KLF2 and upregulates Runx3 expression; 11) Loss of KLF2 leads to reduced S1PR1 expression, favoring tissue retention; 12) Runx3 enhances CD103 expression; 13 and 14) Hobit and Blimp-1 cooperate with Runx3 to promote CD103, CD49a, and CD69 expression, consolidating tissue residency; 15) CD8^+^ T cells express chemokine receptors that control tissue migration and morphology; 16) Sustained TCR, TNFα, TGF-β, and IL-33 signaling maintains Bhlhe40 expression, which supports metabolic homeostasis, oxidative stress resistance, and reinforces the TRM transcriptional program.

Altogether, these findings demonstrate that CD8^+^ TRM cell fate is orchestrated by a coordinated transcriptional network. This includes repression of recirculation-related genes (e.g., S1pr1 and CCR7) and upregulation of residency and survival markers (e.g., CD69, CD103, CD49a). T-bet and Eomes downregulation permits adaptation to tissue niches; RUNX3 acts as a master regulator of the TRM program; Hobit and Blimp-1 further suppress TCM-related transcriptional programs; and Bhlhe40 supports metabolic fitness and long-term TRM persistence. These transcriptional checkpoints not only define TRM identity but also represent promising targets for therapeutic manipulation. **Figure 4** presents an integrated schematic of the main signals characterized in the differentiation of CD8 effector cells into TRMs. The signals may vary depending on the tissue type.

## 4 Targeting signals to improve CD8 TRM responses in viral infections for immunotherapy and vaccine development

Understanding the molecular cues that govern TRM generation and maintenance has direct implications for the design of viral vaccines and antiviral immunotherapies. The induction of efficient TRMs can add an additional layer of peripheral protection, helping to contain the infection at barrier sites. Recent research into the activation of precursor cells and the generation of antiviral CD8^+^ TRMs underscores the critical role of APC and MHC: TCR signaling in this process (47, 50, 53). Furthermore, in the context of Influenza A or Polyoma viral infections, CD8^+^ TRMs are better generated in suboptimal TCR stimulation (60, 61). In addition, these Polyomavirus specific TRM cells exhibited higher-affinity TCRs compared to circulating memory T cells, and once established, antigen presence was no longer essential for TRM retention (59). Based on these observations, it can be inferred that antiviral CD8^+^ TRMs generation was facilitated upon lower TCR stimulation, and the elevated TCR affinity of these cells enhances their ability to detect infected cells, including even those that expressed low levels of antigen. In vaccine or immunotherapeutic strategies aimed at expanding CD8^+^ TRMs, approaches less dependent on antigen quantity may be advantageous. Instead, optimizing antigen delivery and release within the tissue microenvironment may more effectively promote tissue-specific immunity.

One promising approach to enhance CD8^+^ TRMs in mucosal tissues involves the use of chemokines. In mouse models of genital HSV-2 infection, topical administration of CXCL9 and CXCL10 induced migration of CXCR3^+^ CD8^+^ T cells into the vaginal mucosa, where they differentiated into long-lived TRMs that contributed to viral control (146). This strategy could complement conventional vaccines that generate systemic T cell responses, potentially increasing the number of vaginal CD8^+^ TRMs in various genital mucosal sexually transmitted infections. In genital HSV-2 infection, the response to infected cells is modulated by IFN-γ secreted by CD8_+_ TRM lymphocytes (147).

Numerous vaccine models in mouse and immunotherapies for viruses have been extensively studied. These studies highlight the importance of considering key biological factors of lymphocytes—such as activation levels, cytokine responses, and their location within the body—when designing more effective vaccines and immunotherapies to enhance immune responses (148, 149). For example, with mRNA vaccines, the mRNA is engulfed by APCs, and recognized by endosomal and cytosolic innate sensors (150). This recognition triggers cellular activation and a strong generation and programming of antiviral T cells against the cognate antigen (151). The mRNA vaccine has been shown to effectively generate antibody and lymphocyte responses against respiratory pathogens in mice, rabbits and ferrets (152, 153). However, little is known about whether this vaccine model can also induce CD8^+^ TRMs in lung tissue. In a murine model of mRNA vaccination against influenza, the formation of virus specific CD8^+^ TRMs was observed in the lungs when the vaccine was administered either intramuscularly or intranasally. Additionally, prime-boost immunizations in mouse with intramuscular injection followed by intranasal mRNA vaccination led to high levels of both circulating and lung memory T cells against influenza (154). Indeed, while the initial dose can be administered systemically, optimal TRM responses require the booster dose to be delivered directly to the target tissue. This has been demonstrated in experimental mouse models involving genital (155) and lung tissues (156).

The most robust TRM responses are consistently achieved through tissue-targeted stimulation. For instance, immunization via the pulmonary route elicits stronger TRM responses in the lungs, and similar effects are observed when the skin or genital tract is used as the site of immunization. In the ongoing effort to improve vaccine responses to influenza, it was observed that intranasal immunization with S-FLU, a replication-deficient influenza virus vaccine (157), generates specific CD8^+^ TRM lymphocytes in the lung, which are associated with protection. Interestingly, in this model, the characteristic of CD8^+^ TRMs lymphocyte being preferentially generated from clones with lower TCR affinity enhances clonal diversity. This diversity in the receptor allows for the recognition of mutated peptide sequences, which can provide heterosubtypic responses of CD8^+^ TRMs and reduce viral escape (158).

SARS-CoV-2 studies have provided further insights into TRM biology in humans. Individuals who were vaccinated or naturally infected developed nasal CD8^+^ TRMs expressing CD49a that produced IFN-γ upon antigenic stimulation (159). Exploring CD8^+^ TRM signaling might be a useful approach to improving SARS-CoV vaccines. In mice, intranasal vaccination with the viral receptor-binding domain (RBD) combined with polyethyleneimine, a potent mucosal adjuvante for viral glycoprotein antigens (160), demonstrated that this combination significantly expanded specific CD4^+^ and CD8^+^ TRM lymphocytes in lung tissue. These TRMs were bifunctional, producing both IFN-γ and TNF-α, with durability observed in lung tissue for up to one year following intranasal immunization (161). In humans, although the mRNA vaccine against SARS-CoV-2 induced TRM populations in lung tissue, wild type virus elicited a more robust response. The mRNA vaccine generated TRM cells CD69^+^CD103^+^ cells which produced only IFN-γ and failed to persist for very long time in the tissue. However, during infection, the TRMs phenotype were CD69^+^CD103^+^, produced IFN-γ, expressed CD107a, and exhibited a longer lifespan. An optimized lung TRM immune response in humans was observed when vaccination occurred after SARS-Cov 2 infection (162).

Immunization or immunotherapy strategies that target the signals responsible for generating and maintaining CD8 TRM lymphocytes should be prioritized for investigation and development. TRMs are strategically positioned for optimal protection, programmed to mount a rapid response, and can exhibit long-lasting persistence in tissues. These characteristics represent the gold standard for achieving a more effective and durable protective immune response. Perhaps we can deliver antigens to target the TRM response and combining cytokines or chemokines to activate, expand or recruit more cells into the tissue or even use transcription factors modulators in TRMs. However, what remains unknown is how and to what extent TRMs respond to the signals previously characterized for TCM and TEM cells, such as for expression of IL-2, IL-15, IL-21 and IL-7 receptors, CD4-help inside the tissue, cross-presentation and dependence of cytokine for differentiation. Further research is needed to understand how to tailor the TRM response to optimally protect against various pathogens.

## 5 The presence and functionality of TRMs in tumoral microenvironment: perspectives for immunotherapy improvement

More recently, TRMs have become the focus of several studies in tumor immunology. Historically, tumor-infiltrating lymphocytes (TILs) were associated with favorable prognosis. However, as molecular characterization of TILs advanced, it became evident that their differentiation state was a critical determinant of clinical outcomes. While memory T cells (163) were strongly associated with protection and good prognosis, T reg cells (164) and exhausted cells (165) are more associated with poor survival and unfavorable outcomes.

Among the protective memory TILs, CD8^+^CD103^+^ T cells have emerged as the subset most consistently associated with beneficial outcomes across diverse tumor types. In non–small cell lung cancer (NSCLC), increased intraepithelial lymphocyte infiltration and higher patient survival correlated with an expanded CD103^+^ TIL population (166). In that study, freshly isolated CD8^+^CD103^+^ TILs from NSCLC specimens displayed transcriptomic and phenotypic profiles characteristic of TRMs. Similar associations with favorable prognosis were subsequently observed in other NSCLC studies (167, 168), and also verified in human ovarian tumors (169) as well as in human breast tumors (170). Beyond its role in maintaining TRMs within tissues, CD103 is essential for the efficient adhesion of activated T cells to cancer cells. CD103-expressing T cells can lyse tumor cells expressing its ligand, E-cadherin (171). Interestingly, CD8^+^CD103^+^ TRMs often accumulate within fibrotic or stromal regions distant from the tumor epithelium, as seen in pancreatic cancer, suggesting that fibrosis and stromal architecture may physically restrict TRM access, functioning as a tumor immune-evasion mechanism (172).

In most, if not all, of the above-cited studies in humans, protective TRMs frequently express PD-1 and Tim-3 checkpoint receptors. Early single-cell transcriptomic analyses categorized TILs with this signature as exhausted (173–175). More recently, it has become clear that PD-1^+^ TRMs represent the subset most closely associated not only with tumor protection but also with responsiveness to PD-1/PD-L1 checkpoint blockade in humans (174). In murine models, this TIL subset exhibits enhanced activation-induced cell death and mediates potent cytolytic activity toward autologous tumor cells upon blockade of PD-1–PD-L1 interaction (176).

### 5.1 Signals from tumoral microenvironment to TRMs

Similar to virus-infected tissues, CD8^+^ TRMs in tumors must integrate multiple environmental cues to sustain their persistence and antitumor function. Among these, antigen recognition plays a central role in driving TRM activation and differentiation (177). TCR repertoire analyses in human colorectal and gastric cancers show that TIL clonotypes are oligoclonal and enriched for tumor-associated antigens (178, 179). However, many early studies did not distinguish TRMs from other TIL subsets. More recent deep-sequencing data demonstrate that TRM-enriched lesions in melanoma, glioblastoma, and head and neck cancers display distinct TCR diversity shaped by the local antigenic landscape (180–182). Transcription factors governing tissue residency and long-term immune surveillance regulate gene programs that sustain TRM persistence and effector capacity within tumors (183). Additionally, the metabolic milieu of the tumor microenvironment critically influences TRM activity, depending on nutrient availability and hypoxic stress (178). In this section, we will discuss these key factors, highlighting their collective impact on the biology of intratumoral TRMs and their role in shaping the antitumor immune response.

Intratumoral effector CD8^+^ T cells receive antigen-driven signals through TCR stimulation, which promotes their differentiation into CD8^+^ TRMs. Initially, these findings were described in human pancreatic tumors (171). Subsequently, it was demonstrated that the expression of CD103/β7 in human colon carcinoma-specific CTLs is synergistically enhanced by the simultaneous stimulation of TGF-β1 and antigen recognition (179). Antigen dependency of CD8^+^ TRM function was also confirmed in murine brain tumor models and human malignant glioma samples. Functionally, these cells were restimulated as effectors and release IFN-γ and granzyme B (184). Additionally, highly expanded T cell clones tend to accumulate within the tumor microenvironment, leading to a more restricted and oligoclonal TCR repertoire compared to peripheral T cells (182). However, these studies categorize TRMs as part of the broader TIL population without specifically distinguishing them as a unique subset. This lack of differentiation limits our understanding of the specific contributions of TRMs to tumor immunity.

On the other hand, metastatic melanoma lesions in human were observed to be enriched with TRMs, suggesting their involvement in localized immune responses within tumor sites. Notably, these lesions exhibit intralesional TCR diversity, as revealed by deep sequencing of sorted cells for the TCRβ locus. This diversity is likely driven by differences in tumor mutations or neoepitope landscapes among metastases (180). Similar findings also were reported in other malignancies, including human head and neck cancers (185), mouse glioblastoma (186), human vaginal melanoma (187), human oral cavity squamous cell carcinoma (188), and human multiple primary lung cancers (189). Additionally, TRM clonotypes originally identified in tumors were detected in the skin and blood of metastatic melanoma survivors for up to nine years post-treatment (190). Collectively, these studies reinforce the concept that TRMs are key orchestrators of local antitumor immunity and exhibit remarkable adaptability to distinct tumor antigenic environments.

In addition to the TCR in antigen recognition by TRMs, the CD103 molecule plays a fundamental role in enabling these cells to combat tumor growth effectively. Accordingly, CD103 is recruited to the immunological synapse, and its interaction with E-cadherin is essential for cytolytic granule polarization and subsequent exocytosis, leading to efficient tumor cell lysis by tumor TILs. Mechanistically, CD103 ligation triggers phosphorylation of PLCγ1 and ERK1/2, which orchestrate actin remodeling and directed secretion of granzyme and perforin. Inhibiting PLCγ blocks granule relocalization, thereby reducing TCR-mediated cytotoxicity (191). Notably, the interaction between αEβ7 on TILs and E-cadherin on autologous CCL3-producing tumor cells facilitates CCR5 recruitment at the immunological synapse. Moreover, the chemokines CCL3 and CCL5 selectively drive the accumulation of CD103^+^ CD8^+^ T cells, a mechanism observed in human NSCLC (192). Notably, CD103 expression is induced on CD8^+^ T cells following TCR engagement and exposure to TGF-β1, with the transcription factors Smad2/3 and nuclear factor of activated T cells 1 (NFAT-1) serving as critical regulators of this process (193).

Beyond CD103, the CXCR6–CXCL16 axis is emerging as a key determinant of TRM retention, positioning, and survival in tumors (194, 195). CXCR6 deficiency leads to defective TRM recruitment, impaired vaccine responses, and reduced survival in mouse models of head and neck and lung tumors (196). n a mouse pancreatic cancer model, transduction of CXCR6 into CAR-T cells targeting mesothelin (CART-MSLN) promoted tumor rejection and durable remission (197). CXCR6 expression correlates with effector transcriptional programs underscoring the antitumor activity of TRMs in mice (198). Conversely, CXCR6-deficient T cells display increased apoptosis, with gene-set enrichment analyses revealing downregulation of the CD28 signaling pathway, further confirmed by flow cytometry (199). CXCL16, the ligand of CXCR6, plays a pivotal role in T-cell retention and is primarily expressed by antigen-presenting cells (200). However, tumor cells can also express CXCL16 (201) and high levels of this chemokine in tumor cells correlated with increased T-cell infiltration and prolonged patient survival in colorectal cancer (202). Importantly, engineering CAR-T cells to express CXCR6 or RUNX3 enhances their intratumoral persistence and tumor control, highlighting the translational potential of modulating this pathway (203, 204).

Cytokines also serve as critical signaling factors for TRMs. In bladder cancer, TRMs can be activated by anti-CD3, anti-CD28, and IL-15 stimulation, leading to proliferation and increased T-bet, perforin, and granzyme B expression, particularly in the PD-1^+^ subset (205). The importance of IL-15 in cancer-associated TRMs has also been demonstrated in melanoma, where intratumoral IL-15 expression varies among patients and shows a positive correlation with TRM abundance. Furthermore, IL-15 expression is strongly associated with increased mRNA levels of CD8^+^ TRM-related markers, including ITGAE (CD103), ITGA1 (CD49a), and CD69, reinforcing its role in shaping the intratumoral T cell landscape (206). IL-15 plays a central role in sustaining TRM function and cytotoxicity within tumors. TRMs isolated from bladder cancer patients proliferate and upregulate T-bet, perforin, and granzyme B in response to IL-15 stimulation (205). In melanoma, IL-15 expression positively correlates with TRM abundance and higher mRNA levels of ITGAE (CD103), ITGA1 (CD49a), and CD69, reinforcing its role in maintaining residency and effector potential (206). These findings suggest that IL-15 plays a crucial role in sustaining TRM function and cytotoxic potential within the tumor microenvironment.

### 5.2 Perspectives for advanced TRM based immunotherapies for cancer

Recently, several immunotherapeutic strategies and clinical trials targeting CD8^+^ TRMs have been explored. One such approach is Chimeric Antigen Receptor T-cell (CAR-T) therapy, a personalized treatment in which patient-derived lymphocytes are isolated, genetically modified to express a chimeric antigen receptor (CAR), and then re-infused into the patient to enhance tumor cytotoxicity (207). A preclinical study using a liver cancer model demonstrated that overexpression of Runx3 in CAR-T cells significantly improved both the persistence and the infiltration of T cells within tumor tissues. This modification not only enhanced the antitumor activity of CAR-T cells but also contributed to prolonged therapeutic efficacy, offering potential for sustained response in solid tumors (208).

Radiotherapy remains a critical modality in the treatment of solid tumors. While T lymphocytes are radiosensitive, a study in a murine model of colon adenocarcinoma revealed that pre-existing intratumoral T lymphocytes can survive radiotherapy. Notably, the tumor microenvironment appears to reprogram these lymphocytes, inducing a molecular signature similar to that of TRMs. This includes increased expression of CD49a and E-cadherin, along with a reduction in S1P1R expression. These findings suggest the potential for combining radiotherapy with immunotherapy strategies targeting TRMs to enhance therapeutic outcomes (209).

Vaccination models and strategies designed to induce the generation of CD8^+^ TRM lymphocytes specific to tumor antigens are actively being investigated and have demonstrated promising results *in vivo*. For instance, breast cancer, a leading cause of cancer-related mortality worldwide, has a propensity to metastasize the lungs. A nasal mucosa vaccine model utilizing an adenoviral vector encoding tumor-associated antigens demonstrated that both prophylactic and therapeutic applications significantly increased the number of CD8^+^ TRMs in lung tissue. This increase was directly correlated with enhanced control of pulmonary breast cancer metastasis in the prophylactic setting. Furthermore, when combined with radiotherapy, the vaccine synergistically enhanced the relationship between TRMs and therapeutic efficacy, highlighting the potential for combining vaccination and radiotherapy as an integrated approach to cancer treatment (210). Similarly, systemic prime-intranasal boost immunization also provided protection against breast cancer metastasis to the lung. In this model, CD8^+^ TRMs exhibited polyfunctional capacity, secreting key cytokines such as IFN-γ, IL-2, and TNF-α, further underscoring their pivotal role in antitumor immunity (156). Vaccine models of TRM induction also were effective in cutaneous melanoma (211) and HPV-induced carcinomas in the skin and liver (212).

Mechanistic dissection of these pathways has prompted efforts to develop a standardized “TRM immunoscore” that integrates phenotypic composition (CD103, CD69, CD49a) with spatial localization relative to tumor epithelium and vasculature. This framework may help distinguish prognostic biomarkers (linked to survival) from predictive biomarkers (linked to therapeutic response) (213, 214). However, technical limitations remain, such as marker loss during enzymatic dissociation and variable sample processing, which may underestimate TRM frequencies. Standardized protocols and spatially resolved transcriptomic tools will be essential for accurate TRM profiling. Altogether, tumor-resident CD8^+^ TRMs act as dynamic epithelial sentinels that integrate antigenic, adhesive, and cytokine cues to maintain residency and sustain cytotoxic competence, representing a critical target for next-generation immunotherapies.

## 6 Conclusion

This review highlights the pivotal role of TRMs in both immunity to infections and cancer immunotherapy. It emphasizes the unique differentiation and functional properties of TRMs. These cells are formed from differentiated effector CD8^+^ T cells and possess the distinct advantage of residing permanently in tissues. This enables them to provide rapid, localized responses upon re-infection or tumor detection. Their formation is influenced by a complex interplay of antigen recognition, tissue-specific cytokine signals, and transcriptional regulation, with key markers such as CD69, CD103, and CD49a ensuring their retention and function in tissues.

The comparison between viral infections and cancer environments reveals both commonalities and challenges. While the mechanisms of TRM formation share similarities across these contexts, cancer microenvironments present additional immunosuppressive signals that complicate the maintenance of effective TRM responses. The presence of checkpoint receptors such as PD-1 and Tim-3 on TRMs in tumors is indicative of the complex balance between protection and immune evasion. This points to the potential benefits of combining TRM-targeted immunotherapies with immune checkpoint blockade.

The use of CD8^+^ TRM lymphocytes as therapies for infections and cancer faces several challenges. The effective generation of these cells depends on local stimuli, whereas most immunization and immunotherapy strategies still rely on systemic routes, such as intramuscular or intravenous administration. Another obstacle is the limited understanding of the plasticity among memory T cell subpopulations, particularly regarding the potential conversion between TCMs and TRMs, a critical aspect for the regulation of immunological memory. Moreover, much of the current knowledge is derived from murine models, while human studies remain in pre-clinical stages. The difficulty in isolating and manipulating TRMs ex vivo also poses a significant technical limitation. The expression of the PD-1 marker, involved in the functional regulation of TRM cells, remains under investigation in various contexts, particularly regarding its role in the response of these cells to TGF-β (215). Finally, the local activation of these cells may trigger exacerbated inflammatory responses, raising safety concerns. The formation, maintenance, and function of TRMs still depend on molecular and environmental signals that are not yet fully understood, limiting their safe and effective clinical application.

In summary, the regulation of CD8^+^ TRM lymphocytes is a cornerstone of immune responses, providing long-term protection against infections and playing a crucial role in cancer immunotherapy and vaccine optimization. The emerging strategies that aim to enhance TRM function, through either vaccine optimization or immunotherapy, hold great promise for improving the durability and specificity of immune responses, contributing to more effective treatments for both infectious diseases and cancer. Further research into TRM biology is essential for refining these therapeutic strategies and realizing their full potential in clinical settings.

## References

[1] Mestecky J Strober W Russell MW Cheroutre H Lambrecht B Kelsall B . (Eds.) Mucosal Immunology (4th ed.). Academic Press (Elsevier). (2015).

[2] Tomasi Tb Tan Em Solomon A Prendergast Ra . Characteristics of an immune system common to certain external secretions. J Exp Med. (1965). doi: 10.1084/jem.121.1.101, PMID: 14253478 PMC2137965

[3] Ogra PL Karzon DT Righthand F MacGillivray M . Immunoglobulin response in serum and secretions after immunization with live and inactivated poliovaccine and natural infection. New Engl J Med. (1968) 279:893–900. doi: 10.1056/NEJM196810242791701, PMID: 20617594

[4] Mintern JD Guillonneau C Carbone FR Doherty PC Turner SJ . Cutting edge: tissue-resident memory CTL down-regulate cytolytic molecule expression following virus clearance. J Immunol. (2007) 179(11):7220–4. doi: 10.4049/jimmunol.179.11.7220, PMID: 18025163

[5] Wakim LM Gebhardt T Heath WR Carbone FR . Cutting edge: local recall responses by memory T cells newly recruited to peripheral nonlymphoid tissues. J Immunol. (2008) 181(9): 5837–41. doi: 10.4049/jimmunol.181.9.5837, PMID: 18941171

[6] Masopust D Vezys V Marzo AL LL . Preferential localization of effector memory cells in nonlymphoid tissue. Sci (1979). (2001) 23:2413–7. doi: 10.1126/science.1058867, PMID: 11264538

[7] Kok L Masopust D Schumacher TN . The precursors of CD8+ tissue resident memory T cells: from lymphoid organs to infected tissues. Nat Rev Immunol. (2022) 22:283–93. doi: 10.1038/s41577-021-00590-3, PMID: 34480118 PMC8415193

[8] Yang K Kallies A . Tissue-specific differentiation of CD8+ resident memory T cells. Trends Immunol. (2021) 42(10):76–890. doi: 10.1016/j.it.2021.08.002, PMID: 34531111

[9] Obers A Poch T Rodrigues G Christo SN Gandolfo LC Fonseca R . Retinoic acid and TGF-β orchestrate organ-specific programs of tissue residency. Immunity. (2024) 57(11):2615–33.e10. doi: 10.1016/j.immuni.2024.09.015, PMID: 39406245

[10] Gebhardt T Wakim LM Eidsmo L Reading PC Heath WR Carbone FR . Memory T cells in nonlymphoid tissue that provide enhanced local immunity during infection with herpes simplex virus. Nat Immunol. (2009) 10:524–30. doi: 10.1038/ni.1718, PMID: 19305395

[11] Masopust D Choo D Vezys V Wherry EJ Duraiswamy J Akondy R . Dynamic T cell migration program provides resident memory within intestinal epithelium. J Exp Med. (2010) 207(3):553–64. doi: 10.1084/jem.20090858, PMID: 20156972 PMC2839151

[12] Jiang X Clark RA Liu L Wagers AJ Fuhlbrigge RC Kupper TS . Skin infection generates non-migratory memory CD8 + T RM cells providing global skin immunity. Nature. (2012) 483:227–31. doi: 10.1038/nature10851, PMID: 22388819 PMC3437663

[13] MacKay LK Rahimpour A Ma JZ Collins N Stock AT Hafon ML . The developmental pathway for CD103+ CD8+ tissue-resident memory T cells of skin. Nat Immunol. (2013) 14, 1294–301. doi: 10.1038/ni.2744, PMID: 24162776

[14] Mueller SN Mackay LK . Tissue-resident memory T cells: Local specialists in immune defence. Nat Rev Immunol. (2016) 16, 79–89. doi: 10.1038/nri.2015.3, PMID: 26688350

[15] Shiow LR Rosen DB Brdičková N Xu Y An J Lanier LL . CD69 acts downstream of interferon-α/β to inhibit S1P 1 and lymphocyte egress from lymphoid organs. Nature. (2006) 440, 540–4. doi: 10.1038/nature04606, PMID: 16525420

[16] Sheridan BS Lefrançois L . Regional and mucosal memory T cells. Nat Immunol. (2011) 12(6):485–91. doi: 10.1038/ni.2029, PMID: 21739671 PMC3224372

[17] Ray SJ Franki SN Pierce RH Dimitrova S Koteliansky V Sprague AG . The collagen binding α1β1 integrin VLA-1 regulates CD8 T cell-mediated immune protection against heterologous influenza infection. Immunity. (2004) 20(2):167–79. doi: 10.1016/s1074-7613(04)00021-4, PMID: 14975239

[18] Teijaro JR Turner D Pham Q Wherry EJ Lefrançois L Farber DL . Cutting edge: tissue-retentive lung memory CD4 T cells mediate optimal protection to respiratory virus infection. J Immunol. (2011) 187(11):5510–4. doi: 10.4049/jimmunol.1102243, PMID: 22058417 PMC3221837

[19] Doherty PC Riberdy JM Belz GT . Quantitative analysis of the CD8+ T-cell response to readily eliminated and persistent viruses. Philos Trans R Soc B: Biol Sci. (2000) 355(1400):1093–101. doi: 10.1098/rstb.2000.0647, PMID: 11186311 PMC1692813

[20] Seder RA Ahmed R . Similarities and differences in CD4+ and CD8+ effector and memory T cell generation. Nat Immunol. (2003). doi: 10.1038/ni969, PMID: 12942084

[21] Chapatte L Colombetti S Cerottini JC Lévy F . Efficient induction of tumor antigen-specific CD8+ memory T cells by recombinant lentivectors. Cancer Res. (2006) 79(18):57–4566. doi: 10.1158/0008-5472.CAN-05-2597, PMID: 16424053

[22] Joshi NS Cui W Chandele A Lee HK Urso DR Hagman J . Inflammation directs memory precursor and short-lived effector CD8+ T cell fates via the graded expression of T-bet transcription factor. Immunity. (2007) 27(2):281–95. doi: 10.1016/j.immuni.2007.07.010, PMID: 17723218 PMC2034442

[23] Solouki S Huang W Elmore J Limper C Huang F August A . TCR signal strength and antigen affinity regulate CD8+ Memory T cells. J Immunol. (2020) 205(5):1217–27. doi: 10.4049/jimmunol.1901167, PMID: 32759295 PMC8104072

[24] Yajima T Yoshihara K Nakazato K Kumabe S Koyasu S Sad S . IL-15 regulates CD8+ T cell contraction during primary infection. J Immunol. (2006) 176(1):507–15. doi: 10.4049/jimmunol.176.1.507, PMID: 16365444

[25] Ichii H Sakamoto A Hatano M Okada S Toyama H Taki S . Role for BcL-6 in the generation and maintenance of memory CD8+ T cells. Nat Immunol. (2002) 3:558–63. doi: 10.1038/ni802, PMID: 12021781

[26] D’Cruz LM Rubinstein MP Goldrath AW . Surviving the crash: Transitioning from effector to memory CD8+ T cell. Semin Immunol. (2009) 21(2):92–8. doi: 10.1016/j.smim.2009.02.002, PMID: 19269192 PMC2671236

[27] Obar JJ Lefranois L . Memory CD8+ T cell differentiation. Ann New York Acad Sci. (2010) 1183:251–66. doi: 10.1111/j.1749-6632.2009.05126.x, PMID: 20146720 PMC2836783

[28] Michelini RH Doedens AL Goldrath AW Hedrick SM . Differentiation of CD8 memory T cells depends on foxo1. J Exp Med. (2013) 210(6):1189–200. doi: 10.1084/jem.20130392, PMID: 23712431 PMC3674697

[29] Jeannet G Boudousquié C Gardiol N Kang J Huelsken J Held W . Essential role of the Wnt pathway effector Tcf-1 for the establishment of functional CD8 T cell memory. Proc Natl Acad Sci U S A. (2010) 107(21):9777–82. doi: 10.1073/pnas.0914127107, PMID: 20457902 PMC2906901

[30] Chen Y Zander R Khatun A Schauder DM Cui W . Transcriptional and epigenetic regulation of effector and memory CD8 T cell differentiation. Front Immunol. (2018) 7(9):2826. doi: 10.3389/fimmu.2018.02826, PMID: 30581433 PMC6292868

[31] Masopust D Kaech SM Wherry EJ Ahmed R . The role of programming in memory T-cell development. Curr Opin Immunol. (2004) 16(2):217–25. doi: 10.1016/j.coi.2004.02.005, PMID: 15023416

[32] Sallusto F Geginat J Lanzavecchia A . Central memory and effector memory T cell subsets: Function, generation, and maintenance. Annu Rev Immunol. (2004) 22:745–63. doi: 10.1146/annurev.immunol.22.012703.104702, PMID: 15032595

[33] Rahimi RA Luster AD . Chemokines: critical regulators of memory T cell development, maintenance, and function. Adv Immunol. (2018) 138:71–98. doi: 10.1016/bs.ai.2018.02.002, PMID: 29731007 PMC6191293

[34] Sallusto F Lenig D Förster R Lipp M Lanzavecchia A . Two subsets of memory T lymphocytes with distinct homing potentials and effector functions. Nature. (1999) 402(6763), 34–8. doi: 10.1038/35005534 10537110

[35] Geginat J Sallusto F Lanzavecchia A . Cytokine-driven proliferation and differentiation of human naive, central memory, and effector memory CD4+ T cells. J Exp Med. (2001) 194(12):1711–20. doi: 10.1084/jem.194.12.1711, PMID: 11748273 PMC2193568

[36] Zoon CK Seitelman E Keller S Graham L Blevins TL Dumur CI . Expansion of melanoma-specific lymphocytes in alternate gamma chain cytokines: Gene expression variances between T cells and T-cell subsets exposed to IL-2 versus IL-7/15. Cancer Gene Ther. (2014) 21(10):441–7. doi: 10.1038/cgt.2014.48, PMID: 25236493 PMC4205215

[37] Samji T Khanna KM . Understanding memory CD8+T cells. Immunol Lett. (2017) 185:32–9. doi: 10.1016/j.imlet.2017.02.012, PMID: 28274794 PMC5508124

[38] Kaech SM Ahmed R . Memory CD8+ T cell differentiation: Initial antigen encounter triggers a developmental program in naïve cells. Nat Immunol. (2001) 2, 415–22. doi: 10.1038/87720, PMID: 11323695 PMC3760150

[39] Mercado R Vijh S Allen SE Kerksiek K Pilip IM Pamer EG . Early programming of T cell populations responding to bacterial infection. J Immunol. (2000) (12):6833–9. doi: 10.4049/jimmunol.165.12.6833, PMID: 11120806

[40] Boyman O Sprent J . The role of interleukin-2 during homeostasis and activation of the immune system. Nat Rev Immunol. (2012) 12, 180–90. doi: 10.1038/nri3156, PMID: 22343569

[41] Lanzavecchia A Sallusto F . Antigen decoding by T lymphocytes: From synapses to fate determination. Nat Immunol. (2001) 2, 487–92. doi: 10.1038/88678, PMID: 11376334

[42] Lai W Yu M Huang MN Okoye F Keegan AD Farber DL . Transcriptional control of rapid recall by memory CD4 T cells. J Immunol. (2011) 187(1):133–40. doi: 10.4049/jimmunol.1002742, PMID: 21642544 PMC3131107

[43] Ahmed R Gray D . Immunological memory and protective immunity: Understanding their relation. Sci (1979). (1996) 272(5258):54–60. doi: 10.1126/science.272.5258.54, PMID: 8600537

[44] Muroyama Y Wherry EJ . Memory t-cell heterogeneity and terminology. Cold Spring Harb Perspect Med. (2021) 13(10):a037929. doi: 10.1101/cshperspect.a037929, PMID: 33782027 PMC8485749

[45] Lanzavecchia A Sallusto F . Understanding the generation and function of memory T cell subsets. Curr Opin Immunol. (2005) 17(3):326–32. doi: 10.1016/j.coi.2005.04.010, PMID: 15886125

[46] Northrop JK Shen H . CD8+ T-cell memory: Only the good ones last. Curr Opin Immunol. (2004) 16(4):451–5. doi: 10.1016/j.coi.2004.05.004, PMID: 15245738

[47] Muschaweckh A Buchholz VR Fellenzer A Hessel C König PA Tao S . Antigen-dependent competition shapes the local repertoire of tissue-resident memory CD8+ T cells. J Exp Med. (2016) 213(13):3075–86. doi: 10.1084/jem.20160888, PMID: 27899444 PMC5154944

[48] Khan TN Mooster JL Kilgore AM Osborn JF Nolz JC . Local antigen in nonlymphoid tissue promotes resident memory CD8+ T cell formation during viral infection. J Exp Med. (2016) 213(6):951–6. doi: 10.1084/jem.20151855, PMID: 27217536 PMC4886364

[49] Iijima N Iwasaki A . Tissue instruction for migration and retention of TRM cells. Trends Immunol. (2015) 36(9):556–64. doi: 10.1016/j.it.2015.07.002, PMID: 26282885 PMC4567393

[50] Iborra S Martínez-López M Khouili SC Enamorado M Cueto FJ Conde-Garrosa R . Optimal Generation of Tissue-Resident but Not Circulating Memory T Cells during Viral Infection Requires Crosspriming by DNGR-1+ Dendritic Cells. Immunity. (2016) 45(4):847–60. doi: 10.1016/j.immuni.2016.08.019, PMID: 27692611 PMC5074364

[51] Enamorado M Khouili SC Iborra S Sancho D . Genealogy, dendritic cell priming, and differentiation of tissue-resident memory CD8+ T cells. Front Immunol. (2018) 9:1751. doi: 10.3389/fimmu.2018.01751, PMID: 30108585 PMC6079237

[52] Haddadi S Thanthrige-Don N Afkhami S Khera A Jeyanathan M Xing Z . Expression and role of VLA-1 in resident memory CD8 T cell responses to respiratory mucosal viral-vectored immunization against tuberculosis. Sci Rep. (2017) 7(1):9525. doi: 10.1038/s41598-017-09909-4, PMID: 28842633 PMC5573413

[53] McMaster SR Wein AN Dunbar PR Hayward SL Cartwright EK Denning TL . Pulmonary antigen encounter regulates the establishment of tissue-resident CD8 memory T cells in the lung airways and parenchyma article. Mucosal Immunol. (2018) 11(4):1071–8. doi: 10.1038/s41385-018-0003-x, PMID: 29453412 PMC6030505

[54] Cheng L Becattini S . Local antigen encounter promotes generation of tissue-resident memory T cells in the large intestine. Mucosal Immunol. (2024) 17:810–24. doi: 10.1016/j.mucimm.2024.05.005, PMID: 38782240

[55] Tieu R Zeng Q Zhao D Zhang G Feizi N Manandhar P . Tissue-resident memory T cell maintenance during antigen persistence requires both cognate antigen and interleukin-15. Sci Immunol. (2023) 8(82):eadd8454. doi: 10.1126/sciimmunol.add8454, PMID: 37083450 PMC10334460

[56] Wakim LM Woodward-Davis A Bevan MJ . Memory T cells persisting within the brain after local infection show functional adaptations to their tissue of residence. Proc Natl Acad Sci U.S.A. (2010) 107(42):17872–9. doi: 10.1073/pnas.1010201107, PMID: 20923878 PMC2964240

[57] Mackay LK Stock AT Ma JZ Jones CM Kent SJ Mueller SN . Long-lived epithelial immunity by tissue-resident memory T (TRM) cells in the absence of persisting local antigen presentation. Proc Natl Acad Sci U.S.A. (2012) 109(18):7037–42. doi: 10.1073/pnas.1202288109, PMID: 22509047 PMC3344960

[58] Davies B Prier JE Jones CM Gebhardt T Carbone FR Mackay LK . Cutting edge: tissue-resident memory T cells generated by multiple immunizations or localized deposition provide enhanced immunity. J Immunol. (2017). doi: 10.4049/jimmunol.1601367, PMID: 28159905

[59] Frost EL Kersh AE Evavold BD Lukacher AE . Cutting edge: resident memory CD8 T cells express high-affinity TCRs. J Immunol. (2015) 195(8):3520–4. doi: 10.4049/jimmunol.1501521, PMID: 26371252 PMC4592826

[60] Maru S Jin G Schell TD Lukacher AE . TCR stimulation strength is inversely associated with establishment of functional brain-resident memory CD8 T cells during persistent viral infection. PloS Pathog. (2017) 13(4):e1006318. doi: 10.1371/journal.ppat.1006318, PMID: 28410427 PMC5406018

[61] Fiege JK Stone IA Fay EJ Markman MW Wijeyesinghe S Macchietto MG . The impact of TCR signal strength on resident memory T cell formation during influenza virus infection. J Immunol. (2019) 13(2):a037978. doi: 10.4049/jimmunol.1900093, PMID: 31235552 PMC6684852

[62] Abdelbary M Hobbs SJ Gibbs JS Yewdell JW Nolz JC . T cell receptor signaling strength establishes the chemotactic properties of effector CD8+ T cells that control tissue-residency. Nat Commun. (2023) 14(1):3928. doi: 10.1038/s41467-023-39592-1, PMID: 37402742 PMC10319879

[63] Fernandez-Ruiz D Ng WY Holz LE Ma JZ Zaid A Wong YC . Liver-resident memory CD8+ T cells form a front-line defense against malaria liver-stage infection. Immunity. (2019) 51(4):780. doi: 10.1016/j.immuni.2019.09.019, PMID: 31618655

[64] Olsen TM Stone BC Chuenchob V Murphy SC . Prime-and-trap malaria vaccination to generate protective CD8+ Liver-resident memory T cells. J Immunol. (2018) 201(7):1984–93. doi: 10.4049/jimmunol.1800740, PMID: 30127085

[65] Dravid P Murthy S Attia Z Cassady C Chandra R Trivedi S . Phenotype and fate of liver-resident CD8 T cells during acute and chronic hepacivirus infection. PloS Pathog. (2023) 19(10):e1011697. doi: 10.1371/journal.ppat.1011697, PMID: 37812637 PMC10602381

[66] Koelle DM Dong L Jing L Laing KJ Zhu J Jin L . HSV-2-specific human female reproductive tract tissue resident memory T cells recognize diverse HSV antigens. Front Immunol. (2022) 13:1–16. doi: 10.3389/fimmu.2022.867962, PMID: 35432373 PMC9009524

[67] Sanecka A Yoshida N Kolawole EM Patel H Evavold BD Frickel EM . T cell receptor-major histocompatibility complex interaction strength defines trafficking and CD103+ memory status of CD8 T cells in the brain. Front Immunol. (2018) 9:1290. doi: 10.3389/fimmu.2018.01290, PMID: 29922298 PMC5996069

[68] Zheng MZM Wakim LM . Tissue resident memory T cells in the respiratory tract. Mucosal Immunol. (2022) 15(3):379–88. doi: 10.1038/s41385-021-00461-z, PMID: 34671115 PMC8526531

[69] Zhang M Li N He Y Shi T Jie Z . Pulmonary resident memory T cells in respiratory virus infection and their inspiration on therapeutic strategies. Front Immunol. (2022) 13:943331. doi: 10.3389/fimmu.2022.943331, PMID: 36032142 PMC9412965

[70] Szabo PA Miron M Farber DL . Location, location, location: Tissue resident memory T cells in mice and humans. Sci Immunol. (2019) 4(34):eaas9673. doi: 10.1126/sciimmunol.aas9673, PMID: 30952804 PMC6778482

[71] Christo SN Evrard M Park SL Gandolfo LC Burn TN Fonseca R . Discrete tissue microenvironments instruct diversity in resident memory T cell function and plasticity. Nat Immunol. (2021) 22(9):1140–51. doi: 10.1038/s41590-021-01004-1, PMID: 34426691

[72] Kurd NS He Z Louis TL Milner JJ Omilusik KD Jin W . Early precursors and molecular determinants of tissue-resident memory CD8+ T lymphocytes revealed by single-cell RNA sequencing. Sci Immunol. (2020) 5(47):eaaz6894. doi: 10.1126/sciimmunol.aaz6894, PMID: 32414833 PMC7341730

[73] Whitley SK Li M Kashem SW Hirai T Igyártó BZ Knizner K . Local IL-23 is required for proliferation and retention of skin-resident memory TH17 cells. Sci Immunol. (2022) 7(77):eabq325. doi: 10.1126/sciimmunol.abq3254, PMID: 36367947 PMC9847353

[74] Kawasaki T Ikegawa M Yunoki K Otani H Ori D Ishii KJ . Alveolar macrophages instruct CD8+ T cell expansion by antigen cross-presentation in lung. Cell Rep. (2022) 41(11):111828. doi: 10.1016/j.celrep.2022.111828, PMID: 36516765

[75] Crowl JT Heeg M Ferry A Milner JJ Omilusik KD Toma C . Tissue-resident memory CD8+ T cells possess unique transcriptional, epigenetic and functional adaptations to different tissue environments. Nat Immunol. (2022) 23(7):1121–31. doi: 10.1038/s41590-022-01229-8, PMID: 35761084 PMC10041538

[76] González-Amaro R Cortés JR Sánchez-Madrid F Martín P . Is CD69 an effective brake to control inflammatory diseases? Trends Mol Med. (2013) 19(10):625–32. doi: 10.1016/j.molmed.2013.07.006, PMID: 23954168 PMC4171681

[77] Cibrián D Sánchez-Madrid F . CD69: from activation marker to metabolic gatekeeper. Eur J Immunol. (2017) 47(6):946–53. doi: 10.1002/eji.201646837, PMID: 28475283 PMC6485631

[78] Bankovich AJ Shiow LR Cyster JG . CD69 suppresses sphingosine 1-phosophate receptor-1 (S1P1) function through interaction with membrane helix 4. J Biol Chem. (2010) 285(29):22328–37. doi: 10.1074/jbc.M110.123299, PMID: 20463015 PMC2903414

[79] Mackay LK Braun A Macleod BL Collins N Tebartz C Bedoui S . Cutting edge: CD69 interference with sphingosine-1-phosphate receptor function regulates peripheral T cell retention. J Immunol. (2015) 194:2059–63. doi: 10.4049/jimmunol.1402256, PMID: 25624457

[80] Kim JP Chen JD Wilke MS Schall TJ Woodley DT . Human keratinocyte migration on type IV collagen: Roles of heparin- binding site and α2β1 integrin. Lab Invest. (1994) 156(2):424–37., PMID: 7933990

[81] Bank I Book M Ware R . Functional role of VLA-1 (CD49A) in adhesion, cation-dependent spreading, and activation of cultured human T lymphocytes. Cell Immunol. (1994) 156(2):424–37. doi: 10.1006/cimm.1994.1187, PMID: 8025956

[82] Bromley SK Akbaba H Mani V Mora-Buch R Chasse AY Sama A . CD49a regulates cutaneous resident memory CD8+ T cell persistence and response. Cell Rep. (2020) 32(9):108085. doi: 10.1016/j.celrep.2020.108085, PMID: 32877667 PMC7520726

[83] Damei I Trickovic T Mami-Chouaib F Corgnac S . Tumor-resident memory T cells as a biomarker of the response to cancer immunotherapy. Front Immunol. (2023) 14:1205984. doi: 10.3389/fimmu.2023.1205984, PMID: 37545498 PMC10399960

[84] Reilly EC Emo KL Buckley PM Reilly NS Chaves FA Yang H . TRM integrins CD103 and CD49a differentially support adherence and motility after resolution of influenza virus infection. bioRxiv. (2020) 117(22):12306–14. doi: 10.1073/pnas.1915681117, PMID: 32439709 PMC7275699

[85] Hoffmann JC Schön MP . Integrin αe(Cd103)β7 in epithelial cancer. Cancers. (2021) 13(24):6211. doi: 10.3390/cancers13246211, PMID: 34944831 PMC8699740

[86] Romagnoli G D’Alessandris QG Capone I Tavilla A Canini I Lapenta C . CD8+CD103+PD1+TIM3+ T cells in glioblastoma microenvironment correlate with prognosis. Immunology. (2024) 171(2):198–211. doi: 10.1111/imm.13710, PMID: 37884280

[87] Kim Y Shin Y Kang GH . Prognostic significance of CD103+ immune cells in solid tumor: a systemic review and meta-analysis. Sci Rep. (2019) 9(1):3808. doi: 10.1038/s41598-019-40527-4, PMID: 30846807 PMC6405906

[88] Wang ZQ Milne K Derocher H Webb JR Nelson BH Watson PH . CD103 and intratumoral immune response in breast cancer. Clin Cancer Res. (2016) 22(24):6290–7. doi: 10.1158/1078-0432.CCR-16-0732, PMID: 27267849

[89] Mackay LK Wynne-Jones E Freestone D Pellicci DG Mielke LA Newman DM . T-box transcription factors combine with the cytokines TGF-β and IL-15 to control tissue-resident memory T cell fate. Immunity. (2015). doi: 10.1016/j.immuni.2015.11.008, PMID: 26682984

[90] Malenica I Adam J Corgnac S Mezquita L Auclin E Damei I . Integrin-αV-mediated activation of TGF-β regulates anti-tumour CD8 T cell immunity and response to PD-1 blockade. Nat Commun. (2021) 12(1):5209. doi: 10.1038/s41467-021-25322-y, PMID: 34471106 PMC8410945

[91] Huang Y Zhou L Zhang H Zhang L Xi X Sun Y . BMDCs induce the generation of the CD103+CD8+ tissue-resident memory T cell subtype, which amplifies local tumor control in the genital tract. Cell Immunol. (2022) 374:104502. doi: 10.1016/j.cellimm.2022.104502, PMID: 35306373

[92] Walsh DA Borges da Silva H Beura LK Peng C Hamilton SE Masopust D . The functional requirement for CD69 in establishment of resident memory CD8+ T cells varies with tissue location. J Immunol. (2019) 203(4):946–55. doi: 10.4049/jimmunol.1900052, PMID: 31243092 PMC6684481

[93] Kumar BV Ma W Miron M Granot T Guyer RS Carpenter DJ . Human tissue-resident memory T cells are defined by core transcriptional and functional signatures in lymphoid and mucosal sites. Cell Rep. (2017) 20(12):2921–34. doi: 10.1016/j.celrep.2017.08.078, PMID: 28930685 PMC5646692

[94] Liao W Liu Y Ma C Wang L Li G Mishra S . The downregulation of IL-18R defines bona fide kidney-resident CD8+ T cells. iScience. (2021) 24(1):101975. doi: 10.1016/j.isci.2020.101975, PMID: 33474536 PMC7803637

[95] Steinbach K Vincenti I Kreutzfeldt M Page N Muschaweckh A Wagner I . Brain-resident memory T cells represent an autonomous cytotoxic barrier to viral infection. J Exp Med. (2016) 213(8):1571–87. doi: 10.1084/jem.20151916, PMID: 27377586 PMC4986533

[96] Qiu Z Khairallah C Chu TH Imperato JN Lei X Romanov G . Retinoic acid signaling during priming licenses intestinal CD103+ CD8 TRM cell differentiation. J Exp Med. (2023) 220(5):e20210923. doi: 10.1084/jem.20210923, PMID: 36809399 PMC9960115

[97] Cheuk S Schlums H Gallais Sérézal I Martini E Chiang SC Marquardt N . CD49a expression defines tissue-resident CD8+ T cells poised for cytotoxic function in human skin. Immunity. (2017) 46(2):287–300. doi: 10.1016/j.immuni.2017.01.009, PMID: 28214226 PMC5337619

[98] Lund JM Hladik F Prlic M . Advances and challenges in studying the tissue-resident T cell compartment in the human female reproductive tract. Immunol Rev. (2023) 316(1):52–62. doi: 10.1111/imr.13212, PMID: 37140024 PMC10524394

[99] Kamimura D Bevan MJ . Naive CD8+ T cells differentiate into protective memory-like cells after IL-2-anti-IL-2 complex treatment *in vivo*. J Exp Med. (2007) 204(8):1803–12. doi: 10.1084/jem.20070543, PMID: 17664293 PMC2118678

[100] Murali-Krishna K Altman JD Suresh M Sourdive DJD Zajac AJ Miller JD . Counting antigen-specific CD8 T cells: A reevaluation of bystander activation during viral infection. Immunity. (1998) 8(2):177–87. doi: 10.1016/S1074-7613(00)80470-7, PMID: 9491999

[101] Zaid A Mackay LK Rahimpour A Braun A Veldhoen M Carbone FR . Persistence of skin-resident memory T cells within an epidermal niche. Proc Natl Acad Sci U.S.A. (2014) 111(14):5307–12. doi: 10.1073/pnas.1322292111, PMID: 24706879 PMC3986170

[102] Chen WJ . TGF-β Regulation of T cells. Annu Rev Immunol. (2023) 41:483–512. doi: 10.1146/annurev-immunol-101921-045939, PMID: 36750317 PMC12453633

[103] Zhang N Bevan MJ . Transforming growth factor-β signaling controls the formation and maintenance of gut-resident memory T cells by regulating migration and retention. Immunity. (2013) 39(4):687–96. doi: 10.1016/j.immuni.2013.08.019, PMID: 24076049 PMC3805703

[104] Casey KA Fraser KA Schenkel JM Moran A Abt MC Beura LK . Antigen-independent differentiation and maintenance of effector-like resident memory T cells in tissues. J Immunol. (2012) 188(10):4866–75. doi: 10.4049/jimmunol.1200402, PMID: 22504644 PMC3345065

[105] Ma C Mishra S Demel EL Liu Y Zhang N . TGF-β Controls the formation of kidney-resident T cells via promoting effector T cell extravasation. J Immunol. (2017) 198(2):749–56. doi: 10.4049/jimmunol.1601500, PMID: 27903738 PMC5225110

[106] Schenkel JM Fraser KA Casey KA Beura LK Pauken KE Vezys V . IL-15–independent maintenance of tissue-resident and boosted effector memory CD8 T cells. J Immunol. (2016) 196(9):3920–6. doi: 10.4049/jimmunol.1502337, PMID: 27001957 PMC5145194

[107] Jarjour NN Wanhainen KM Peng C Gavil NV Maurice NJ da Silva HB . Responsiveness to interleukin-15 therapy is shared between tissue-resident and circulating memory CD8+ T cell subsets. Proc Natl Acad Sci U.S.A. (2022) 119(43):e2209021119. doi: 10.1073/pnas.2209021119, PMID: 36260745 PMC9618124

[108] Jarjour NN Dalzell TS Maurice NJ Wanhainen KM Peng C DePauw TA . Collaboration between IL-7 and IL-15 enables adaptation of tissue-resident and circulating memory CD8(+) T cells. Immunity. (2025) 58:616–31. doi: 10.1016/j.immuni.2025.02.009, PMID: 40023156 PMC13329436

[109] Tian Y Cox MA Kahan SM Ingram JT Bakshi RK Zajac AJ . A context-dependent role for IL-21 in modulating the differentiation, distribution, and abundance of effector and memory CD8 T cell subsets. J Immunol. (2016) 119(43):e2209021119. doi: 10.4049/jimmunol.1401236, PMID: 26826252 PMC4761492

[110] Ren HM Kolawole EM Ren M Jin G Netherby-Winslow CS Wade Q . IL-21 from high-affinity CD4 T cells drives differentiation of brain-resident CD8 T cells during persistent viral infection. Sci Immunol. (2020) 5(51):eabb5590. doi: 10.1126/sciimmunol.abb5590, PMID: 32948671 PMC7721466

[111] Marchesini Tovar G Espinal AM Gallen C BT . IL-33 increases the magnitude of the tissue-resident memory T cell response in intestinal tissues during local infection. Jounral Immunol. (2024) 2013:1887–92. doi: 10.4049/jimmunol.2400323, PMID: 39465972 PMC12178136

[112] Ariotti S Beltman JB Chodaczek G Hoekstra ME Van Beek AE Gomez-Eerland R . Tissue-resident memory CD8+ T cells continuously patrol skin epithelia to quickly recognize local antigen. Proc Natl Acad Sci U.S.A. (2012) 109(48):19739–44. doi: 10.1073/pnas.1208927109, PMID: 23150545 PMC3511734

[113] Zaid A Hor JL Christo SN Groom JR Heath WR Mackay LK . Chemokine receptor–dependent control of skin tissue–resident memory T cell formation. J Immunol. (2017) 199(7):2451–9. doi: 10.4049/jimmunol.1700571, PMID: 28855310

[114] Homey B Alenius H Müller A Soto H Bowman EP Yuan W . CCL27-CCR10 interactions regulate T cell-mediated skin inflammation. Nat Med. (2002) 8(2):157–65. doi: 10.1038/nm0202-157, PMID: 11821900

[115] Bromley SK Mempel TR Luster AD . Orchestrating the orchestrators: Chemokines in control of T cell traffic. Nat Immunol. (2008) 9(9):970–80. doi: 10.1038/ni.f.213, PMID: 18711434

[116] Van Braeckel-Budimir N Varga SM Badovinac VP Harty JT . Repeated antigen exposure extends the durability of influenza-specific lung-resident memory CD8+ T cells and heterosubtypic immunity. Cell Rep. (2018) 24(13):3374–82.e3. doi: 10.1016/j.celrep.2018.08.073, PMID: 30257199 PMC6258017

[117] Schenkel JM Fraser KA Vezys V Masopust D . Sensing and alarm function of resident memory CD8 + T cells. Nat Immunol. (2013) 14(5):509–13. doi: 10.1038/ni.2568, PMID: 23542740 PMC3631432

[118] McCully ML Ladell K Andrews R Jones RE Miners KL Roger L . CCR8 expression defines tissue-resident memory T cells in human skin. J Immunol. (2018) 200(5):1639–50. doi: 10.4049/jimmunol.1701377, PMID: 29427415 PMC5818732

[119] Ritzel RM Crapser J Patel AR Verma R Grenier JM Chauhan A . Age-associated resident memory CD8 T cells in the central nervous system are primed to potentiate inflammation after ischemic brain injury. J Immunol. (2016) 196(8):3318–30. doi: 10.4049/jimmunol.1502021, PMID: 26962232 PMC4868658

[120] Srivastava R Hernández-Ruiz M Khan AA Fouladi MA Kim GJ Ly VT . CXCL17 chemokine–dependent mobilization of CXCR8 + CD8 + Effector memory and tissue-resident memory T cells in the vaginal mucosa is associated with protection against genital herpes. J Immunol. (2018) 200:2915–26. doi: 10.4049/jimmunol.1701474, PMID: 29549178 PMC5893430

[121] Caldeira-Dantas S Furmanak T Smith C Quinn M Teos LY Ertel A . The Chemokine Receptor CXCR3 Promotes CD8+ T Cell Accumulation in Uninfected Salivary Glands but Is Not Necessary after Murine Cytomegalovirus Infection. J Immunol. (2018) 200(3):1133–45. doi: 10.4049/jimmunol.1701272, PMID: 29288198 PMC5780235

[122] Srivastava R Khan AA Chilukuri S Syed SA Tran TT Furness J . CXCL10/CXCR3-Dependent Mobilization of Herpes Simplex Virus-Specific CD8 + T EM and CD8 + T RM Cells within Infected Tissues Allows Efficient Protection against Recurrent Herpesvirus Infection and Disease. J Virol. (2017) 91(14):e00278-17. doi: 10.1128/JVI.00278-17, PMID: 28468883 PMC5487556

[123] Wein AN McMaster SR Takamura S Dunbar PR Cartwright EK Hayward SL . CXCR6 regulates localization of tissue-resident memory CD8 T cells to the airways. J Exp Med. (2019) 216(12):2748–62. doi: 10.1084/jem.20181308, PMID: 31558615 PMC6888981

[124] Takamura S Kato S Motozono C Shimaoka T Ueha S Matsuo K . Interstitial-resident memory CD8+ T cells sustain frontline epithelial memory in the lung. J Exp Med. (2019) 216(12):2736–47. doi: 10.1084/jem.20190557, PMID: 31558614 PMC6888985

[125] Milner JJ Goldrath AW . Transcriptional programming of tissue-resident memory CD8 + T cells. Curr Opin Immunol. (2018) 51:162–9. doi: 10.1016/j.coi.2018.03.017, PMID: 29621697 PMC5943164

[126] Behr FM Kragten NAM Wesselink TH Nota B Van Lier RAW Amsen D . Blimp-1 rather than hobit drives the formation of tissue-resident memory CD8+T cells in the lungs. Front Immunol. (2019) 10:400. doi: 10.3389/fimmu.2019.00400, PMID: 30899267 PMC6416215

[127] Milner JJ Toma C He Z Kurd NS Nguyen QP McDonald B . Heterogenous populations of tissue-resident CD8+ T cells are generated in response to infection and Malignancy. Immunity. (2020) 111(14):5307–12. doi: 10.1016/j.immuni.2020.04.007, PMID: 32433949 PMC7784612

[128] Skon CN Lee JY Anderson KG Masopust D Hogquist KA Jameson SC . Transcriptional downregulation of S1pr1 is required for the establishment of resident memory CD8+ T cells. Nat Immunol. (2013) 14(12):1285–93. doi: 10.1038/ni.2745, PMID: 24162775 PMC3844557

[129] Pipkin ME Sacks JA Cruz-Guilloty F Lichtenheld MG Bevan MJ Rao A . Interleukin-2 and inflammation induce distinct transcriptional programs that promote the differentiation of effector cytolytic T cells. Immunity. (2010) 32(1):79–90. doi: 10.1016/j.immuni.2009.11.012, PMID: 20096607 PMC2906224

[130] Banerjee A Gordon SM Intlekofer AM Paley MA Mooney EC Lindsten T . Cutting edge: the transcription factor eomesodermin enables CD8+ T cells to compete for the memory cell niche. J Immunol. (2010) 185(9):4988–92. doi: 10.4049/jimmunol.1002042, PMID: 20935204 PMC2975552

[131] Joshi NS Cui W Dominguez CX Chen JH Hand TW Kaech SM . Increased numbers of preexisting memory CD8 T cells and decreased T-bet expression can restrain terminal differentiation of secondary effector and memory CD8 T cells. J Immunol. (2011) 187(8):4068–76. doi: 10.4049/jimmunol.1002145, PMID: 21930973 PMC3991478

[132] Pritchard GH Phan AT Christian DA Blain TJ Fang Q Johnson J . Early T-bet promotes LFA1 upregulation required for CD8+ effector and memory T cell development. J Exp Med. (2023) 220(2):e20191287. doi: 10.1084/jem.20191287, PMID: 36445307 PMC9712775

[133] Laidlaw BJ Zhang N Marshall HD Staron MM Guan T Hu Y . CD4+ T cell help guides formation of CD103+ Lung-resident memory CD8+ T cells during influenza viral infection. Immunity. (2014) 41(4):633–45. doi: 10.1016/j.immuni.2014.09.007, PMID: 25308332 PMC4324721

[134] van Aalderen MC Remmerswaal EBM Verstegen NJM Hombrink P ten Brinke A Pircher H . Infection history determines the differentiation state of human CD8 + T cells. J Virol. (2015) 89(9):5110–23. doi: 10.1128/JVI.03478-14, PMID: 25717102 PMC4403462

[135] Toska A Modi N Chen LF . RUNX3 meets the ubiquitin-proteasome system in cancer. Cells. (2023) 12(5):717. doi: 10.3390/cells12050717, PMID: 36899853 PMC10001085

[136] Chuang LSH Ito Y . RUNX3 is multifunctional in carcinogenesis of multiple solid tumors. Oncogene. (2010) 29(18):2605–15. doi: 10.1038/onc.2010.88, PMID: 20348954

[137] Zitti B Hoffer E Zheng W Pandey RV Schlums H Perinetti Casoni G . Human skin-resident CD8+ T cells require RUNX2 and RUNX3 for induction of cytotoxicity and expression of the integrin CD49a. Immunity. (2023) 56(6):1285–302.e7. doi: 10.1016/j.immuni.2023.05.003, PMID: 37269830

[138] Milner JJ Toma C Yu B Zhang K Omilusik K Phan AT . Runx3 programs CD8+ T cell residency in non-lymphoid tissues and tumours. Nature. (2017) 552(7684):253–7. doi: 10.1038/nature24993, PMID: 29211713 PMC5747964

[139] Fonseca R Burn TN Gandolfo LC Devi S Park SL Obers A . Runx3 drives a CD8+ T cell tissue residency program that is absent in CD4+ T cells. Nat Immunol. (2022) 23(8):1236–45. doi: 10.1038/s41590-022-01273-4, PMID: 35882933 PMC13045866

[140] Mackay LK Minnich M Kragten NAM Liao Y Nota B Seillet C . Hobit and Blimp1 instruct a universal transcriptional program of tissue residency in lymphocytes. Sci (1979). (2016) 352(6284):459–63. doi: 10.1126/science.aad2035, PMID: 27102484

[141] Parga-Vidal L Taggenbrock RLRE Beumer-Chuwonpad A Aglmous H Kragten NAM Behr FM . Hobit and Blimp-1 regulate TRM abundance after LCMV infection by suppressing tissue exit pathways of TRMprecursors. Eur J Immunol. (2022) 52(7):1095–111. doi: 10.1002/eji.202149665, PMID: 35389518 PMC9545210

[142] Park SL Mackay LK . Bhlhe40 keeps resident T cells too fit to quit. Immunity. (2019) 51(3):418–20. doi: 10.1016/j.immuni.2019.08.016, PMID: 31533052

[143] Lin CC Bradstreet TR Schwarzkopf EA Sim J Carrero JA Chou C . Bhlhe40 controls cytokine production by T cells and is essential for pathogenicity in autoimmune neuroinflammation. Nat Commun. (2014) 5:3551. doi: 10.1038/ncomms4551, PMID: 24699451 PMC4016562

[144] Cook ME Jarjour NN Lin CC Edelson BT . Transcription factor bhlhe40 in immunity and autoimmunity. Trends Immunol. (2020) 41(11):1023–36. doi: 10.1016/j.it.2020.09.002, PMID: 33039338 PMC7606821

[145] Li C Zhu B Son YM Wang Z Jiang L Xiang M . The transcription factor bhlhe40 programs mitochondrial regulation of resident CD8+ T cell fitness and functionality. Immunity. (2019) 51(3):491–507.e7. doi: 10.1016/j.immuni.2019.08.013, PMID: 31533057 PMC6903704

[146] Shin H Iwasaki A . A vaccine strategy that protects against genital herpes by establishing local memory T cells. Nature. (2012) 491(7424):463–7. doi: 10.1038/nature11522, PMID: 23075848 PMC3499630

[147] Shin H Kumamoto Y Gopinath S Iwasaki A . CD301b+ dendritic cells stimulate tissue-resident memory CD8+ T cells to protect against genital HSV-2. Nat Commun. (2016) 7:13346. doi: 10.1038/ncomms13346, PMID: 27827367 PMC5105190

[148] Sallusto F Lanzavecchia A Araki K Ahmed R . Immunity review from vaccines to memory and back. Immun Rev. (2010). doi: 10.1016/j.immuni.2010.10.008, PMID: 21029957 PMC3760154

[149] Pulendran B Ahmed R . Immunological mechanisms of vaccination. Nat Immunol. (2011) 33(4):451–63. doi: 10.1038/ni.2039, PMID: 21739679 PMC3253344

[150] Gote V Bolla PK Kommineni N Butreddy A Nukala PK Palakurthi SS . A comprehensive review of mRNA vaccines. Int J Mol Sci. (2023) 24(3):2700. doi: 10.3390/ijms24032700, PMID: 36769023 PMC9917162

[151] Teijaro JR Farber DL . COVID-19 vaccines: modes of immune activation and future challenges. Nat Rev Immunol. (2021) 21(4):195–7. doi: 10.1038/s41577-021-00526-x, PMID: 33674759 PMC7934118

[152] Zhang Z Mateus J Coelho CH Dan JM Moderbacher CR Gálvez RI . Humoral and cellular immune memory to four COVID-19 vaccines. Cell. (2022) 185(14):2434–51.e17. doi: 10.1016/j.cell.2022.05.022, PMID: 35764089 PMC9135677

[153] Pardi N Parkhouse K Kirkpatrick E McMahon M Zost SJ Mui BL . Nucleoside-modified mRNA immunization elicits influenza virus hemagglutinin stalk-specific antibodies. Nat Commun. (2018) 9(1):3361. doi: 10.1038/s41467-018-05482-0, PMID: 30135514 PMC6105651

[154] Künzli M O’Flanagan SD LaRue M Talukder P Dileepan T Stolley JM . Route of self-amplifying mRNA vaccination modulates the establishment of pulmonary resident memory CD8 and CD4 T cells. Sci Immunol. (2022) 7(78):eadd3075. doi: 10.1101/2022.06.02.494574, PMID: 36459542 PMC9832918

[155] Çuburu N Kim R Guittard GC Thompson CD Day PM Hamm DE . A prime-pull-amplify vaccination strategy to maximize induction of circulating and genital-resident intraepithelial CD8 + Memory T cells. J Immunol. (2019) 202:1250–64., PMID: 30635393 10.4049/jimmunol.1800219 PMC6391875

[156] Xu H Yue M Zhou R Wang P Wong MY Wang J . A prime-boost vaccination approach induces lung resident memory CD8+ T cells derived from central memory T cells that prevent tumor lung metastasis. Cancer Res. (2024) 84:3173–88. doi: 10.4049/jimmunol.1800219, PMID: 39350665 PMC11443216

[157] Baz M Boonnak K Paskel M Santos C Powell T Townsend A . Nonreplicating influenza a virus vaccines confer broad protection against lethal challenge. mBio. (2015) 6(5):e01487-15. doi: 10.1128/mBio.01487-15, PMID: 26489862 PMC4620468

[158] Zheng MZM Tan TK Villalon-Letelier F Lau H Deng YM Fritzlar S . Single-cycle influenza virus vaccine generates lung CD8+ Trm that cross-react against viral variants and subvert virus escape mutants. Sci Adv. (2023) 9(36):eadg3469. doi: 10.1126/sciadv.adg3469, PMID: 37683004 PMC10491285

[159] Rha MS Kim G Lee S Kim J Jeong Y Jung CM . SARS-CoV-2 spike-specific nasal-resident CD49a+CD8+ memory T cells exert immediate effector functions with enhanced IFN-γ production. Nat Commun. (2024) 27:8355. doi: 10.1038/s41467-024-52689-5, PMID: 39333516 PMC11436836

[160] Wegmann F Gartlan KH Harandi AM Brinckmann SA Coccia M Hillson WR . Polyethyleneimine is a potent mucosal adjuvant for viral glycoprotein antigens. Nat Biotechnol. (2012) 30(9):883–8. doi: 10.1038/nbt.2344, PMID: 22922673 PMC3496939

[161] Lei H Alu A Yang J Ren W He C Lan T . Intranasal administration of a recombinant RBD vaccine induces long-term immunity against Omicron-included SARS-CoV-2 variants. Signal Transduct Target Ther. (2022) 7(1):159. doi: 10.1038/s41392-022-01002-1, PMID: 35581200 PMC9112270

[162] Pieren DKJ Kuguel SG Rosado J Robles AG Rey-Cano J Mancebo C . Limited induction of polyfunctional lung-resident memory T cells against SARS-CoV-2 by mRNA vaccination compared to infection. Nat Commun. (2023) 14(1):1887. doi: 10.1038/s41467-023-37559-w, PMID: 37019909 PMC10074357

[163] Pagès F Galon J Dieu-Nosjean MC Tartour E Sautès-Fridman C Fridman WH . Immune infiltration in human tumors: A prognostic factor that should not be ignored. Oncogene. (2010) 29(8):1093–102. doi: 10.1038/onc.2009.416, PMID: 19946335

[164] Ghebeh H Barhoush E Tulbah A Elkum N Al-Tweigeri T Dermime S . FOXP3+ Tregs and B7-H1+/PD-1+ T lymphocytes co-infiltrate the tumor tissues of high-risk breast cancer patients: Implication for immunotherapy. BMC Cancer. (2008) 8:57. doi: 10.1186/1471-2407-8-57, PMID: 18294387 PMC2279136

[165] Kim PS Ahmed R . Features of responding T cells in cancer and chronic infection. Curr Opin Immunol. (2010) 22(2):223–30. doi: 10.1016/j.coi.2010.02.005, PMID: 20207527 PMC2892208

[166] Djenidi F Adam J Goubar A Durgeau A Meurice G de Montpréville V . CD8+CD103+ Tumor–infiltrating lymphocytes are tumor-specific tissue-resident memory T cells and a prognostic factor for survival in lung cancer patients. J Immunol. (2015) 194(7):3475–86. doi: 10.4049/jimmunol.1402711, PMID: 25725111

[167] Oja AE Piet B van der Zwan D Blaauwgeers H Mensink M De Kivit S . Functional heterogeneity of CD4+ tumor-infiltrating lymphocytes with a resident memory phenotype in NSCLC. Front Immunol. (2018) 9:2654. doi: 10.3389/fimmu.2018.02654, PMID: 30505306 PMC6250821

[168] Corgnac S Malenica I Mezquita L Auclin E Voilin E Kacher J . CD103+CD8+ TRM cells accumulate in tumors of anti-PD-1-responder lung cancer patients and are tumor-reactive lymphocytes enriched with tc17. Cell Rep Med. (2020) 1(7):100127. doi: 10.1016/j.xcrm.2020.100127, PMID: 33205076 PMC7659589

[169] Webb JR Milne K Nelson BH . PD-1 and CD103 are widely coexpressed on prognostically favorable intraepithelial CD8 T cells in human ovarian cancer. Cancer Immunol Res. (2015) 3(8):926–35. doi: 10.1158/2326-6066.CIR-14-0239, PMID: 25957117

[170] Savas P Virassamy B Ye C Salim A Mintoff CP Caramia F . Single-cell profiling of breast cancer T cells reveals a tissue-resident memory subset associated with improved prognosis. Nat Med. (2018) 24(12):1941. doi: 10.1038/s41591-018-0176-6, PMID: 30135555

[171] Ademmer K Ebert M Müller-Ostermeyer F Friess H Büchler MW Schubert W . Effector T lymphocyte subsets in human pancreatic cancer: Detection of CD8+ CD18+ cells and CD8+ CD103+ cells by multi-epitope imaging. Clin Exp Immunol. (1998) 112(1):21–6. doi: 10.1046/j.1365-2249.1998.00546.x, PMID: 9566785 PMC1904939

[172] French JJ Cresswell J Wong WK Seymour K Charnley RM Kirby JA . T cell adhesion and cytolysis of pancreatic cancer cells: A role for E-cadherin in immunotherapy? Br J Cancer. (2002) 87(9):1034–41. doi: 10.1038/sj.bjc.6600597, PMID: 12434297 PMC2364324

[173] Clarke J Panwar B Madrigal A Singh D Gujar R Wood O . Single-cell transcriptomic analysis of tissue-resident memory T cells in human lung cancer. J Exp Med. (2019) 216(9):2128–49. doi: 10.1084/jem.20190249, PMID: 31227543 PMC6719422

[174] Banchereau R Chitre AS Scherl A Wu TD Patil NS De Almeida P . Intratumoral CD103+ CD8+ T cells predict response to PD-L1 blockade. J Immunother Cancer. (2021) 9(4):e002231. doi: 10.1136/jitc-2020-002231, PMID: 33827905 PMC8032254

[175] Guo L Cao C Goswami S Huang X Ma L Guo Y . Tumoral PD-1hiCD8+ T cells are partially exhausted and predict favorable outcome in triple-negative breast cancer. Clin Sci. (2020) 134(7):711–26. doi: 10.1042/CS20191261, PMID: 32202617

[176] Fernandez-Poma SM Salas-Benito D Lozano T Casares N Riezu-Boj JI Mancheño U . Expansion of tumor-infiltrating CD8+ T cells expressing PD-1 improves the efficacy of adoptive T-cell therapy. Cancer Res. (2017) 77(13):3672–84. doi: 10.1158/0008-5472.CAN-17-0236, PMID: 28522749

[177] Wei CH Trenney R Sanchez-Alavez M Marquardt K Woodland DL Henriksen SJ . Tissue-resident memory CD8+ T cells can be deleted by soluble, but not cross-presented antigen. J Immunol. (2005) 175(10):6615–23. doi: 10.4049/jimmunol.175.10.6615, PMID: 16272316

[178] Lin R Zhang H Yuan Y He Q Zhou J Li S . Fatty acid oxidation controls CD8+Tissue-resident memory t-cell survival in gastric adenocarcinoma. Cancer Immunol Res. (2020) 8(4):479–92. doi: 10.1158/2326-6066.CIR-19-0702, PMID: 32075801

[179] Ling KL Dulphy N Bahl P Salio M Maskell K Piris J . Modulation of CD103 expression on human colon carcinoma-specific CTL. J Immunol. (2007) 178(5):2908–15. doi: 10.4049/jimmunol.178.5.2908, PMID: 17312135

[180] Boddupalli CS Bar N Kadaveru K Krauthammer M Pornputtapong N Mai Z . Interlesional diversity of T cell receptors in melanoma with immune checkpoints enriched in tissue-resident memory T cells. JCI Insight. (2016) 1(21):e88955. doi: 10.1172/jci.insight.88955, PMID: 28018970 PMC5161225

[181] Jia Q Zhou J Chen G Shi Y Yu H Guan P . Diversity index of mucosal resident T lymphocyte repertoire predicts clinical prognosis in gastric cancer. Oncoimmunology. (2015) 4(4):e1001230. doi: 10.1080/2162402X.2014.1001230, PMID: 26137399 PMC4485732

[182] Sherwood AM Emerson RO Scherer D Habermann N Buck K Staffa J . Tumor-infiltrating lymphocytes in colorectal tumors display a diversity of T cell receptor sequences that differ from the T cells in adjacent mucosal tissue. Cancer Immunology Immunotherapy. (2013) 62(9):1453–61. doi: 10.1007/s00262-013-1446-2, PMID: 23771160 PMC5714653

[183] Wakim LM Woodward-Davis A Liu R Hu Y Villadangos J Smyth G . The molecular signature of tissue resident memory CD8 T cells isolated from the brain. J Immunol. (2012) 189(7):3462–71. doi: 10.4049/jimmunol.1201305, PMID: 22922816 PMC3884813

[184] Masson F Calzascia T Di Berardino-Besson W de Tribolet N Dietrich PY Walker PR . Brain microenvironment promotes the final functional maturation of tumor-specific effector CD8+ T cells. J Immunol. (2008) 179(2):845–53. doi: 10.4049/jimmunol.179.2.845, PMID: 17617575

[185] Oliveira G Egloff AM Afeyan AB Wolff JO Zeng Z Chernock RD . Preexisting tumor-resident T cells with cytotoxic potential associate with response to neoadjuvant anti-PD-1 in head and neck cancer. Sci Immunol. (2023) 8(87):eadf4968. doi: 10.1126/sciimmunol.adf4968, PMID: 37683037 PMC10794154

[186] Zhao Q Hu J Kong L Jiang S Tian X Wang J . FGL2-targeting T cells exhibit antitumor effects on glioblastoma and recruit tumor-specific brain-resident memory T cells. Nat Commun. (2023) 14(1):735. doi: 10.1038/s41467-023-36430-2, PMID: 36759517 PMC9911733

[187] Pizzolla A Keam SP Vergara IA Caramia F Thio N Wang M . Tissue-resident memory T cells from a metastatic vaginal melanoma patient are tumor-responsive T cells and increase after anti-PD-1 treatment. J Immunother Cancer. (2022) 10(5):e004574. doi: 10.1136/jitc-2022-004574, PMID: 35550554 PMC9109124

[188] Luoma AM Suo S Wang Y Gunasti L Porter CBM Nabilsi N . Tissue-resident memory and circulating T cells are early responders to pre-surgical cancer immunotherapy. Cell. (2022) 185(16):2918–35.e29. doi: 10.1016/j.cell.2022.06.018, PMID: 35803260 PMC9508682

[189] Zhang C Yin K Liu SY Yan LX Su J Wu YL . Multiomics analysis reveals a distinct response mechanism in multiple primary lung adenocarcinoma after neoadjuvant immunotherapy. J Immunother Cancer. (2021) 9(4):e002312. doi: 10.1136/jitc-2020-002312, PMID: 33820821 PMC8025811

[190] Han J Zhao Y Shirai K Molodtsov A Kolling FW Fisher JL . Resident and circulating memory T cells persist for years in melanoma patients with durable responses to immunotherapy. Nat Cancer. (2021) 9(4):e002312. doi: 10.1038/s43018-021-00180-1, PMID: 34179824 PMC8223731

[191] Le Floc’h A Jalil A Franciszkiewicz K Validire P Vergnon I Mami-Chouaib F . Minimal engagement of CD103 on cytotoxic T lymphocytes with an E-cadherin-Fc molecule triggers lytic granule polarization via a phospholipase Cγ-dependent pathway. Cancer Res. (2011) 71(2):328–38. doi: 10.1158/0008-5472.CAN-10-2457, PMID: 21224355

[192] Franciszkiewicz K Le Floc’h A Jalil A Vigant F Robert T Vergnon I . Intratumoral induction of CD103 triggers tumor-specific CTL function and CCR5-dependent T-cell retention. Cancer Res. (2009). doi: 10.1158/0008-5472.CAN-08-3571, PMID: 19638592

[193] Mokrani M Klibi J Bluteau D Bismuth G Mami-Chouaib F . Smad and NFAT pathways cooperate to induce CD103 expression in human CD8 T lymphocytes. J Immunol. (2014) 69(15):6249–55. doi: 10.4049/jimmunol.1302192, PMID: 24477908

[194] Wang B Wang Y Sun X Deng G Huang W Wu X . CXCR6 is required for antitumor efficacy of intratumoral CD8 + T cell. J Immunother Cancer. (2021) 9(8):e003100. doi: 10.1136/jitc-2021-003100, PMID: 34462326 PMC8407215

[195] Muthuswamy R McGray AR Battaglia S He W Miliotto A Eppolito C . CXCR6 by increasing retention of memory CD8 + T cells in the ovarian tumor microenvironment promotes immunosurveillance and control of ovarian cancer. J Immunother Cancer. (2021) 9(10):e003329. doi: 10.1136/jitc-2021-003329, PMID: 34607898 PMC8491420

[196] Karaki S Blanc C Tran T Galy-Fauroux I Mougel A Dransart E . CXCR6 deficiency impairs cancer vaccine efficacy and CD8 + resident memory T-cell recruitment in head and neck and lung tumors. J Immunother Cancer. (2021) 9(3):e001948. doi: 10.1136/jitc-2020-001948, PMID: 33692218 PMC7949477

[197] Lesch S Blumenberg V Stoiber S Gottschlich A Ogonek J Cadilha BL . T cells armed with C-X-C chemokine receptor type 6 enhance adoptive cell therapy for pancreatic tumours. Nat BioMed Eng. (2021) 5(11):1246–60. doi: 10.1038/s41551-021-00737-6, PMID: 34083764 PMC7611996

[198] Di Pilato M Kfuri-Rubens R Pruessmann JN Ozga AJ Messemaker M Cadilha BL . CXCR6 positions cytotoxic T cells to receive critical survival signals in the tumor microenvironment. Cell. (2021) 184(17):4512–30.e22. doi: 10.1016/j.cell.2021.07.015, PMID: 34343496 PMC8719451

[199] Tooley K Jerby L Escobar G Krovi SH Mangani D Dandekar G . Pan-cancer mapping of single CD8+ T cell profiles reveals a TCF1:CXCR6 axis regulating CD28 co-stimulation and anti-tumor immunity. Cell Rep Med. (2024) 5:101640. doi: 10.1016/j.xcrm.2024.101640, PMID: 38959885 PMC11293343

[200] Vella JL Molodtsov A Angeles CV Branchini BR Turk MJ Huang YH . Dendritic cells maintain anti-tumor immunity by positioning CD8 skin-resident memory T cells. Life Sci Alliance. (2021) 4(10):e202101056. doi: 10.26508/lsa.202101056, PMID: 34362825 PMC8356251

[201] Xing YN Xu XY Nie XC Yang X Yu M Xu HM . Role and clinicopathologic significance of CXC chemokine ligand 16 and chemokine (C-X-C motif) receptor 6 expression in gastric carcinomas. Hum Pathol. (2012) 43(12):2299–307. doi: 10.1016/j.humpath.2011.08.027, PMID: 22863086

[202] Hojo S Koizumi K Tsuneyama K Arita Y Cui Z Shinohara K . High-level expression of chemokine CXCL16 by tumor cells correlates with a good prognosis and increased tumor-infiltrating lymphocytes in colorectal cancer. Cancer Res. (2007) 67(10):4725–31. doi: 10.1158/0008-5472.CAN-06-3424, PMID: 17510400

[203] Fu Q Zheng Y Fang W Zhao Q Zhao P Liu L . RUNX-3-expressing CAR T cells targeting glypican-3 in patients with heavily pretreated advanced hepatocellular carcinoma: a phase I trial. EClinicalMedicine. (2023) 63:102175. doi: 10.1016/j.eclinm.2023.102175, PMID: 37680942 PMC10480529

[204] Mabrouk N Tran T Sam I Pourmir I Gruel N Granier C . CXCR6 expressing T cells: Functions and role in the control of tumors. Front Immunol. (2022) 13:1022136. doi: 10.3389/fimmu.2022.1022136, PMID: 36311728 PMC9597613

[205] Hartana CA Ahlén Bergman E Broomé A Berglund S Johansson M Alamdari F . Tissue-resident memory T cells are epigenetically cytotoxic with signs of exhaustion in human urinary bladder cancer. Clin Exp Immunol. (2018) 194(1):39–53. doi: 10.1111/cei.13183, PMID: 30009527 PMC6156818

[206] Edwards J Wilmott JS Madore J Gide TN Quek C Tasker A . CD103+ tumor-resident CD8+ T cells are associated with improved survival in immunotherapy-naïve melanoma patients and expand significantly during anti-PD-1 treatment. Clin Cancer Res. (2018) 24(13):3036–45. doi: 10.1158/1078-0432.CCR-17-2257, PMID: 29599411

[207] Labanieh L Majzner RG Mackall CL . Programming CAR-T cells to kill cancer. Nat Biomed Eng. (2018) 2(6):377–91. doi: 10.1038/s41551-018-0235-9, PMID: 31011197

[208] Wang Y Zhang H Du G Luo H Su J Sun Y . Enforced expression of Runx3 improved CAR-T cell potency in solid tumor via enhancing resistance to activation-induced cell death. Mol Ther. (2023) 31(3):701–14. doi: 10.1016/j.ymthe.2022.12.009, PMID: 36523165 PMC10014350

[209] Arina A Beckett M Fernandez C Zheng W Pitroda S Chmura SJ . Tumor-reprogrammed resident T cells resist radiation to control tumors. Nat Commun. (2019) 10(1):3959. doi: 10.1038/s41467-019-11906-2, PMID: 31477729 PMC6718618

[210] Oltmanns F Antão AV Irrgang P Viherlehto V Jörg L Schmidt A . Mucosal tumor vaccination delivering endogenous tumor antigens protects against pulmonary breast cancer metastases. J Immunother Cancer. (2024) 12(3):e008652. doi: 10.1136/jitc-2023-008652, PMID: 38458636 PMC10921546

[211] Gálvez-Cancino F López E Menares E Díaz X Flores C Cáceres P . Vaccination-induced skin-resident memory CD8+ T cells mediate strong protection against cutaneous melanoma. Oncoimmunology. (2018) 7(7):e1442163. doi: 10.1080/2162402X.2018.1442163, PMID: 29900048 PMC5993487

[212] Van Der Gracht ETI Schoonderwoerd MJA Van Duikeren S Yilmaz AN Behr FM Colston JM . Adenoviral vaccines promote protective tissue-resident memory T cell populations against cancer. J Immunother Cancer. (2020) 8(2):e001133. doi: 10.1136/jitc-2020-001133, PMID: 33293355 PMC7725098

[213] Ramirez DE Mohamed A Huang YH Turk MJ . In the right place at the right time: tissue-resident memory T cells in immunity to cancer. Curr Opin Immunol. (2023) 83:102338. doi: 10.1016/j.coi.2023.102338, PMID: 37229984 PMC10631801

[214] Wu H Fan PW Feng YN Chang C Gui T Meng JB . Role of T cell exhaustion and tissue-resident memory T cells in the expression and prognosis of colorectal cancer. Nat Sci Rep. (2025), 15(1):28503. doi: 10.1038/s41598-025-14409-x, PMID: 40764738 PMC12325763

[215] Devi P Wang E Jaiswal A Kim T Nirschl J Verma A . PD-1 is requisite for skin T(RM) cell formation and specification by TGFbeta. Nat Immunol. (2025) 26(8):1339–51. doi: 10.1038/s41590-025-02228-1, PMID: 40730902 PMC12307224

